# Increased cytosolic calcium buffering contributes to a cellular arrhythmogenic substrate in iPSC-cardiomyocytes from patients with dilated cardiomyopathy

**DOI:** 10.1007/s00395-022-00912-z

**Published:** 2022-05-02

**Authors:** Philipp Jung, Fitzwilliam Seibertz, Funsho E. Fakuade, Nadezda Ignatyeva, Shrivatsan Sampathkumar, Melanie Ritter, Housen Li, Fleur E. Mason, Antje Ebert, Niels Voigt

**Affiliations:** 1 Institute of Pharmacology and Toxicology, University Medical Center Göttingen, Robert-Koch-Straße 40, 37075 Göttingen, Germany; 2 DZHK (German Center for Cardiovascular Research), Partner Site, Göttingen, Germany; 3 Heart Research Center, Department of Cardiology and Pneumology, University Medical Center Göttingen, Robert-Koch-Straße 40, 37075 Göttingen, Germany; 4 Institute for Mathematical Stochastics, Georg-August University, Göttingen, Germany; 5 Cluster of Excellence “Multiscale Bioimaging: from Molecular Machines to Networks of Excitable Cells” (MBExC), University of Göttingen, Göttingen, Germany

**Keywords:** iPSCs, Calcium handling, Cardiomyopathy, Ion channel, Action potential, Cardiovascular

## Abstract

Dilated cardiomyopathy (DCM) is a major risk factor for heart failure and is associated with the development of life-threatening cardiac arrhythmias. Using a patient-specific induced pluripotent stem cell-derived cardiomyocyte (iPSC-CM) model harbouring a mutation in cardiac troponin T (R173W), we aim to examine the cellular basis of arrhythmogenesis in DCM patients with this mutation. iPSC from control (Ctrl) and DCM-TnT-R173W donors from the same family were differentiated into iPSC-CM and analysed through optical action potential (AP) recordings, simultaneous measurement of cytosolic calcium concentration ([Ca^2+^]_i_) and membrane currents and separately assayed using field stimulation to detect the threshold for AP- and [Ca^2+^]_i_-alternans development. AP duration was unaltered in TnT-R173W iPSC-CM. Nevertheless, TnT-R173W iPSC-CM showed a strikingly low stimulation threshold for AP- and [Ca^2+^]_i_-alternans. Myofilaments are known to play a role as intracellular Ca^2+^ buffers and here we show increased Ca^2+^ affinity of intracellular buffers in TnT-R173W cells, indicating increased myofilament sensitivity to Ca^2+^. Similarly, EMD57033, a myofilament Ca^2+^ sensitiser, replicated the abnormal [Ca^2+^]_i_ dynamics observed in TnT-R173W samples and lowered the threshold for alternans development. In contrast, application of a Ca^2+^ desensitiser (blebbistatin) to TnT-R173W iPSC-CM was able to phenotypically rescue Ca^2+^ dynamics, normalising Ca^2+^ transient profile and minimising the occurrence of Ca^2+^ alternans at physiological frequencies. This finding suggests that increased Ca^2+^ buffering likely plays a major arrhythmogenic role in patients with DCM, specifically in those with mutations in cardiac troponin T. In addition, we propose that modulation of myofilament Ca^2+^ sensitivity could be an effective anti-arrhythmic target for pharmacological management of this disease.

**Supplementary Information:**

The online version contains supplementary material available at 10.1007/s00395-022-00912-z.

## Introduction

Dilated cardiomyopathy (DCM) represents the most common cardiomyopathy and is a major contributor to heart failure and sudden cardiac death [41]. Roughly, 40% of DCM cases are caused by inherited mutations, particularly in genes encoding for sarcomeric proteins [18, 42]. More than 50 disease-related genes have been identified, which also include mutations in cytoskeletal, mitochondrial and ion-channel proteins [41]. The lethal complications of DCM are largely due to the increased incidence of cardiac arrhythmias [33]. While previous work heavily focuses on the molecular basis of impaired contractile function, little is known about the mechanisms underlying cardiac arrhythmias in patients with DCM.

Human induced pluripotent stem cell-derived cardiomyocytes (iPSC-CM) are an emerging tool for modelling cardiac disease and for investigating the molecular basis of cardiac arrhythmias [25, 29, 44, 77]. The first mutation reported in a human patient-specific iPSC-CM model of dilated cardiomyopathy (DCM) was troponin T (TnT)-R173W [63]. Impaired calcium (Ca^2+^) handling and reduced contractility are key features of patient-specific iPSC-CM carrying TnT-R173W [39, 63, 76]. Furthermore, it has been shown that TnT-R173W limits binding of protein kinase A to sarcomeric microdomains and attenuates consecutive phosphorylation of sarcomeric proteins such as troponin I (TnI) [9]. Since hypophosphorylation of TnI typically increases Ca^2+^ affinity of sarcomeric troponin C (TnC) [5, 60], it follows that Ca^2+^ buffering by myofilaments would be increased in DCM TnT-R173W iPSC-CM within a certain range of cytosolic Ca^2+^, which can be visualised in the form of a buffer power curve [59].

Interestingly, increased myofilament Ca^2+^ sensitivity has been suggested to promote the occurrence of life-threatening arrhythmias in patients with familial hypertrophic cardiomyopathy (HCM) [1]. In particular, in mice with HCM-causing TnT mutations, the risk of developing ventricular arrhythmias was directly proportional to the degree of Ca^2+^ sensitisation caused by the mutation. We, therefore, hypothesise that a similar mechanism, i.e. increased Ca^2+^ binding to myofilaments, may also contribute to arrhythmogenesis in patients with DCM-causing TnT mutations.

Here, we utilised DCM patient-specific iPSC-CM carrying the TnT-R173W mutation (DCM-TnT-R173W iPSC-CM) to assess whether alterations in cellular Ca^2+^ handling and cellular electrophysiology may contribute to arrhythmogenesis in DCM patients harbouring mutations in TnT.

## Materials and methods

### Cardiac differentiation of human iPSC

Human induced pluripotent stem cells (iPSC) were grown to 80% confluence on Matrigel-coated plates using chemically defined E8 medium [8, 13] (Supplementary Fig. 1A) and were differentiated into beating iPSC-CM via a small molecule-based monolayer method, as described previously [12, 13, 34, 35]. From day 7, beating iPSC-CM could be observed. Following differentiation, human iPSC-CM were cultured in RPMI medium with B-27 Supplement (Life Technologies). TnT-R173W and Ctrl groups expressed regular levels of pluripotency markers in iPSC and cardiac markers in iPSC-CM, respectively (Supplementary Fig. 1). Following 25 days of cardiac differentiation, beating iPSC-CM monolayers were dissociated using TrypLE and plated onto Matrigel-coated glass coverslips (diameter 10 mm). Cells were investigated within a timeframe of 30–40 days after differentiation. Prior to experimentation, cells were loaded with 0.1 × VoltageFluor2.1Cl (Fluovolt, Thermo Scientific; 20 min loading) for Optical action potential (AP) analysis or 10 µM Fluo-3-acetoxymethyl ester (Fluo-3-AM, Thermo Scientific; 10 min loading, 50 min de-esterification) for intracellular Ca^2+^ investigation in a bath solution containing (in mM): CaCl_2_ 2, Glucose 10, HEPES 10, KCl 4, MgCl_2_ 1, NaCl 140, Probenecid 2; pH = 7.35 adjusted with NaOH. All protocols were approved by the Ethics Committee of the University Medical Center Göttingen (No. 10/9/15 and 15/2/20). Informed consent was obtained from all participants and all research was performed in accordance with relevant guidelines and regulations.

### Electrical field stimulation of iPSC-CM

Coverslips containing iPSC-CM were transferred to a 37 ± 0.5 °C heated chamber containing bath solution. Cells were electrically stimulated at increasing frequencies (0.5 Hz, 1 Hz, 2 Hz, 3 Hz, 4 Hz and 5 Hz) with two parallel platinum electrodes connected to an external stimulator (IonOptix Myopacer cell stimulator). Stimuli were set to 3–5 ms bipolar pulses with voltages ~ 25% above the contraction threshold (normally between 10 and 30 V). APs were recorded from isolated masked cells on the stage of an epifluorescence microscope (λ_Ex_ = 470 nm, λ_Em_ = 535 nm), which was optimised for high-speed signal capture with a photomultiplier as previously described [52, 58]. Three APs from each cell at every measured frequency were ensemble averaged for offline analysis of AP parameters with Clampfit 10.7 (Molecular Devices). Whole-trace AP alternans magnitude was analysed using a discrete Fourier transform-based spectral method as described previously [15, 50]. Cytosolic Ca^2+^ levels were estimated as a change in fluorescence intensity relative to the resting fluorescence intensity at the beginning of each experiment (ΔF/F_0_).

### Patch-clamp and simultaneous intracellular Ca^2+^ measurements

Coverslips containing iPSC-CM were transferred to a 37 ± 0.5 °C heated chamber and were superfused with bath solution containing (in mM): 4-aminopyridine 5, BaCl_2_ 0.1, CaCl_2_ 2, Glucose 10, HEPES 10, KCl 4, MgCl_2_ 1, NaCl 140, Probenecid 2; pH = 7.35 adjusted with NaOH. Simultaneous measurements of membrane currents and intracellular Ca^2+^ were performed under voltage-clamp using the whole-cell ruptured-patch configuration. Membrane currents were measured and analysed using pClamp-Software (V 10.7 Molecular Devices). Fluo-3 pentapotassium salt, 0.1 mM (Thermo Scientific) was added to the pipette solution containing (in mM): EGTA 0.02, GTP-Tris 0.1, HEPES 10, K-aspartate 92, KCl 48, Mg-ATP 1, Na_2_-ATP 4; pH = 7.2 adjusted with KOH. Tip resistances of borosilicate glass microelectrodes were 3–7 MΩ. A voltage-clamp protocol using a holding potential of − 80 mV and a 100 ms voltage step to +10 mV at 0.5 Hz was employed to activate L-type Ca^2+^ current (I_Ca,L_) and corresponding triggered Ca^2+^ transients. A 100 ms ramp pulse to − 40 mV to inactivate the fast Na^+^ current was applied before each depolarising step. Membrane capacitance measurements were acquired and current was expressed as current density (pA/pF).

To quantify intracellular Ca^2+^ concentration ([Ca^2+^]_i_), Fluo-3 was excited at 488 nm and emitted light (> 520 nm) converted to [Ca^2+^]_i_, assuming

$$[{\text{Ca}}^{{2 + }} ]_{i} = k_{d} \left( {\frac{F}{{F_{{max}} - F}}} \right),$$

where *k*_*d*_ is the dissociation constant of Fluo-3 (864 nM), *F* is the Fluo-3 fluorescence; *F*_*max*_ is the Ca^2+^-saturated fluorescence obtained at the end of each experiment [70, 71]. Ca^2+^ transients were analysed by averaging 10 consecutive traces. Sarcoplasmic reticulum (SR) Ca^2+^ content and Ca^2+^ buffering were quantified as previously described by the application of high concentration caffeine (10 mM) [10, 14, 59, 66].

Measurements of Ca^2+^ fluxes (integrated I_Ca,L_) and SR Ca^2+^ content (integrated I_NCX_) are expressed per litre total cell volume, which has been estimated based on a capacitance to volume relationship of 4.57 pF/pL [21].

### Statistical analysis

Summarised data are reported as mean ± SEM, unless otherwise specified. Clustering of experimental data within separate differentiations was tested in 6 WT differentiations and appeared to be negligible (Supplementary Fig. 2). Continuous data with a sample size *n* ≥ 30 were assumed to be normally distributed (central limit theorem) [28]. Values with a distribution between *n* = 10–30 were tested for normality using the Shapiro–Wilk test. Normally distributed data were compared using unpaired two-tailed Student’s *t* test. Non-normally distributed data and all data sets with n < 10 were compared using the Mann–Whitney *U* test, as indicated in the figure legends. Kaplan–Meier curve data were compared using the Gehan–Breslow–Wilcoxon test. A *P* value < 0.05 was considered to be statistically significant.

## Results

### Action potential alternans in DCM-TnT-R173W iPSC-CM

**Fig. 1:**
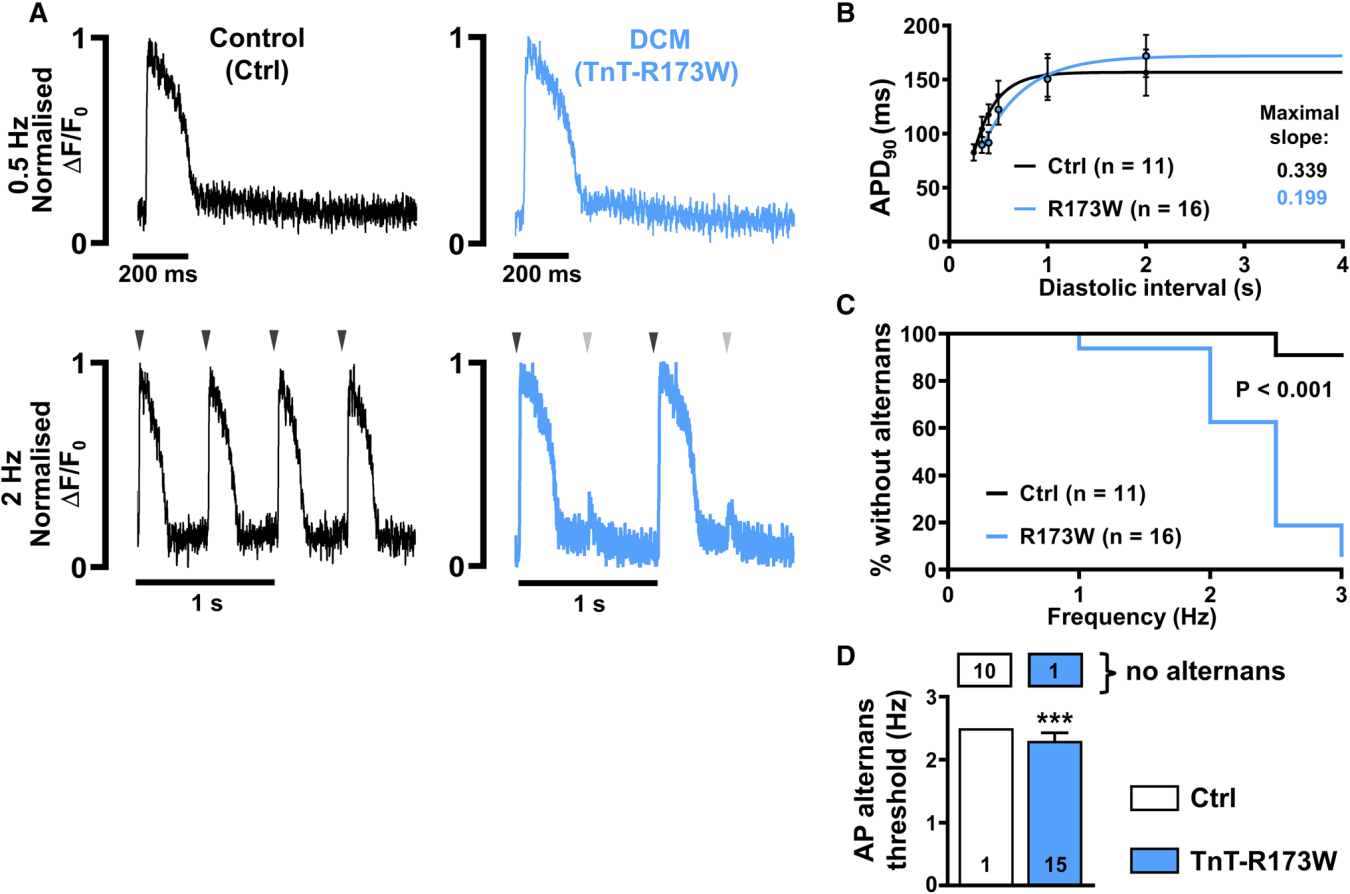
Incidence of action potential (AP) alternans in control (Ctrl) and DCM-TnT-R173W induced pluripotent stem cell-derived cardiomyocytes (iPSC-CM). **A** Normalised representative traces of optical AP at 0.5 Hz (upper) and 2 Hz (lower) in Ctrl (left) and TnT-R173W (right) iPSC-CM. Arrowheads indicate electrical stimulation and illustrate when beat-to-beat alterations are present. **B** Action potential duration at 90% repolarisation (APD_90_) at increasing diastolic intervals (AP restitution) fitted with a one-phase association nonlinear function to determine maximum curve slope. **C** Kaplan–Meier plot showing the percentage of cells without alternans in relation to the respective pacing frequency. **D** Alternans threshold frequency. Number of myocytes without AP alternans is shown in boxes above. *n* = number of iPSC-CM from three batches. Data are mean ± SEM. ***P < 0.001 using the Mann–Whitney *U* test (**D**) and Gehan–Breslow–Wilcoxon test (**C**)

We first assessed optical AP characteristics of isolated iPSC-CM from DCM patients carrying the cardiac troponin T mutation (R173W) and control iPSC-CM from the same family (Ctrl). iPSC-CM were stimulated at a range of frequencies using electrical field stimulation. AP duration at 90% repolarisation (APD_90_) was not significantly different between both groups (Fig. 1A, B, 0.5 Hz: APD_90_ R173W: 171 ± 19.6, n/N = 16/3 vs. Ctrl: 156 ± 21.4 ms, n/N = 11/3). In addition, post-rest potentiation was not enhanced in R173W cells (Supplementary Fig. 3A, B) [57]. AP restitution, describing the relationship between APD and the previous diastolic interval, was also unchanged. Neither group produced a curve with a maximal slope greater than 1 (Fig. 1B). A maximal slope of 1 or greater is assumed to be a pre-requisite for the development of action potential-driven alternans, a phenomenon describing beat-to-beat variation in AP morphology [51, 73]. Despite this, AP alternans was observed at higher frequencies in DCM-TnT-R173W iPSC-CM, and was almost absent in Ctrl iPSC-CM (Fig. 1C, D). A discrete Fourier transform spectral analysis revealed a higher incidence of alternans during both phase 0–1 and phase 2–3 of an action potential event (Supplementary Fig. 3C, D).

### Ca^2+^ alternans in DCM-TnT-R173W iPSC-CM

**Fig. 2:**
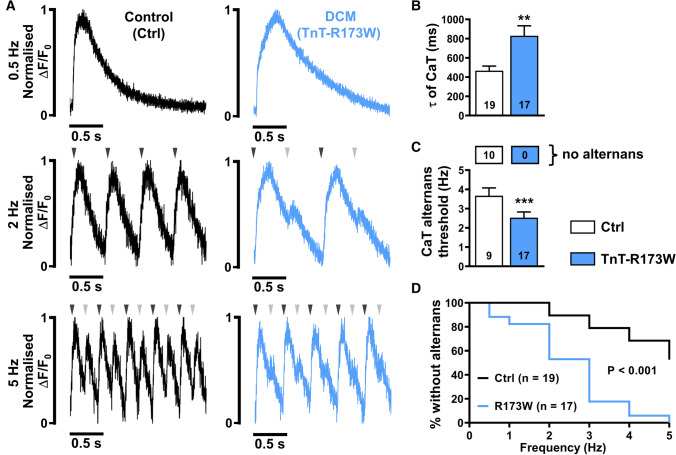
Incidence of Ca^2+^ alternans in control (Ctrl) and DCM-TnT-R173W induced pluripotent stem cell-derived cardiomyocytes (iPSC-CM). **A** Normalised representative traces of Ca^2+^ transients (CaT) at 0.5 Hz (upper), 2 Hz (middle) and 5 Hz (lower) in Ctrl (left) and TnT-R173W (right) iPSC-CM. Arrowheads indicate electrical stimulation and illustrate when beat-to-beat alterations are present. **B** Ca^2+^ transient time constant of decay (τ). **C** Alternans threshold frequency. Number of myocytes without CaT alternans is shown in boxes above. **D** Kaplan–Meier plot showing the percentage of cells without alternans in relation to the respective pacing frequency. *n* = number of iPSC-CM from three batches. Data are mean ± SEM. **P < 0.01 and ***P < 0.001 vs. Ctrl using Student’s *t* test (**B**), Mann–Whitney *U* test (**C**) and the Gehan–Breslow–Wilcoxon test (**D**)

It has been shown previously that DCM-TnT-R173W iPSC-CM are characterised by impaired systolic contractility and slowed diastolic relaxation [9], with the latter pointing to impaired diastolic Ca^2+^ removal from the cytosol [39]. Impaired diastolic Ca^2+^ homeostasis has been shown to contribute to Ca^2+^-driven alternans and, therefore, represent a major mechanism of cardiac arrhythmias. To further investigate diastolic Ca^2+^ handling and potential arrhythmogenic mechanisms, Fluo-3-loaded DCM-TnT-R173W iPSC-CM were stimulated at 0.5 Hz using electrical field stimulation. Representative normalised traces are shown in Fig. 2A (upper panel). Similar to previous studies [9, 39, 63], DCM-TnT-R173W iPSC-CM showed delayed Ca^2+^ transient time-to-peak values (Supplementary Fig. 4A). The time constant of decay was quantified by fitting a single exponential curve to the decay phase of the transient (from 90 to 10% of the amplitude). The time constant of decay was higher in DCM-TnT-R173W iPSC-CM (Fig. 2B), suggesting slower Ca^2+^ removal from the cytosol, which is hypothesised to predispose the cardiomyocyte to the occurrence of Ca^2+^ transient alternans, i.e. beat-to-beat alterations of systolic Ca^2+^ transient amplitude [15, 73].

The occurrence of Ca^2+^ transient alternans in DCM-TnT-R173W iPSC-CM was investigated by increasing stimulation frequency stepwise up to 5 Hz (Fig. 2A). Strikingly, at 5 Hz, Ca^2+^ transient alternans was observed in all DCM-TnT-R173W iPSC-CM but only in 47% of Ctrl iPSC-CM. Kaplan–Meier analysis of alternans occurrence over the whole range of frequencies revealed significantly higher susceptibility to Ca^2+^ transient alternans in R173W-mutant cells. In addition, the threshold for Ca^2+^ transient alternans, i.e. mean frequency at which alternans first occurs, was significantly lower in the DCM-TnT-R173W group (Fig. 2C). Taken together, DCM-TnT-R173W iPSC-CM show slower Ca^2+^ removal from the cytosol, which may contribute to the occurrence of arrhythmogenic alternans in DCM patients harbouring this mutation.

### Smaller amplitude of I_Ca,L_-triggered Ca^2+^ transient in TnT-R173W iPSC-CM

**Fig. 3:**
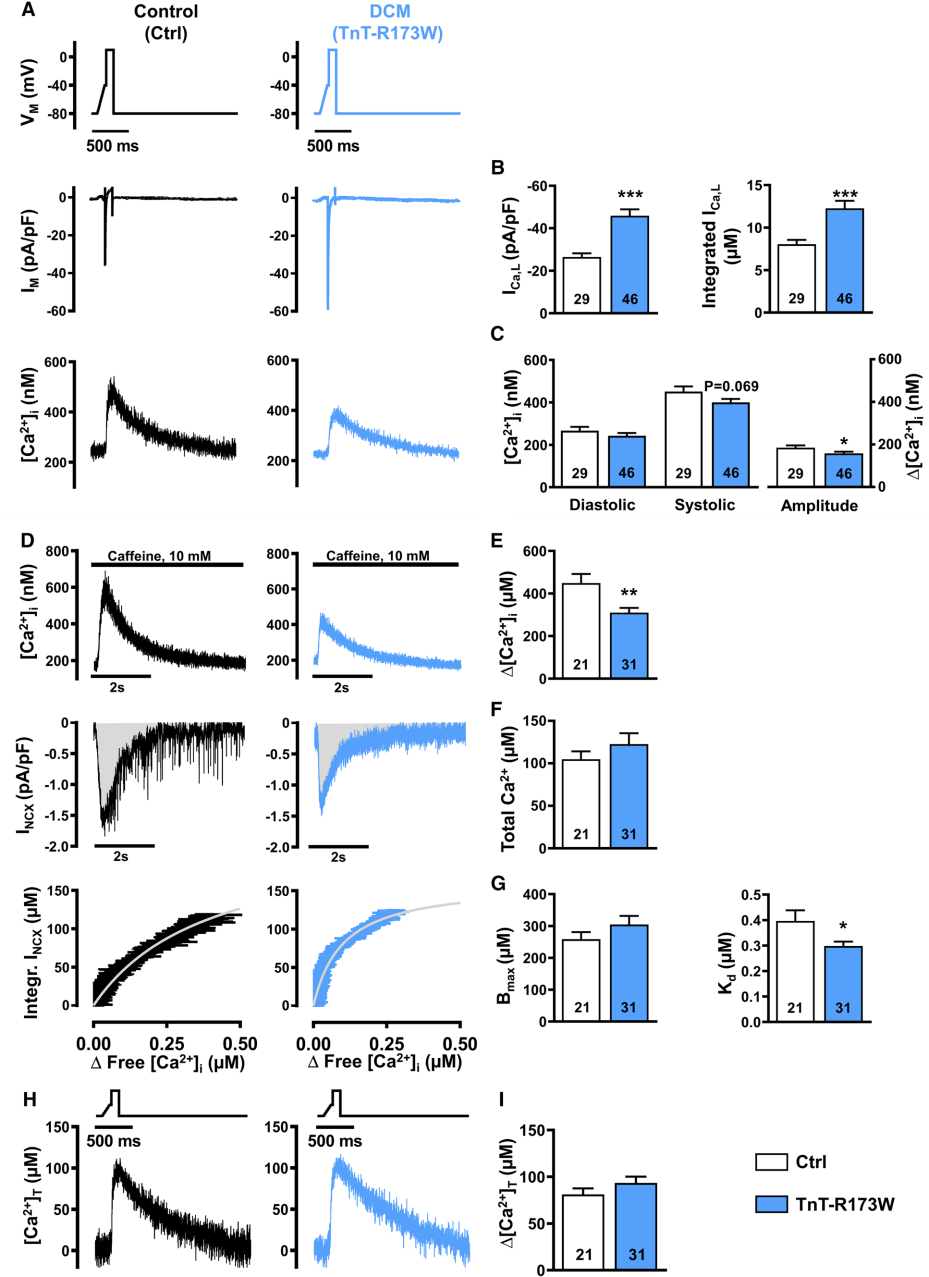
I_Ca,L_ and Ca^2+^ transient (upper), sarcoplasmic reticulum Ca^2+^ load and intracellular Ca^2+^ buffering (middle) and total cytosolic Ca^2+^ concentration during I_Ca,L_ triggering (lower) in control (Ctrl) and DCM-TnT-R173W induced pluripotent stem cell-derived cardiomyocytes (iPSC-CM). **A** Voltage-clamp protocol (upper), representative simultaneous recordings of I_Ca,L_ (middle) and corresponding I_Ca,L_-triggered Ca^2+^ transients (CaT, lower) in Ctrl (left) and TnT-R173W (right) iPSC-CM. **B** Peak I_Ca,L_ amplitude (left) and integrated I_Ca,L_ (right). **C** Diastolic and systolic [Ca^2+^]_i_ (left) and Ca^2+^ transient amplitude (right). **D** Representative recordings of caffeine-induced Ca^2+^ transient, i.e. free cytosolic Ca^2+^ concentration (upper) with associated depolarising inward current (I_NCX_, middle) in Ctrl (left) and TnT-R173W (right) iPSC-CM. Integrated I_NCX_ as an index for total cytosolic Ca^2+^ concentration was plotted against corresponding cytosolic free Ca^2+^ concentration (lower). Buffer curves depicting the relationship between cytosolic free and total Ca^2+^ were fitted with hyperbolic functions. **E**, **F** Sarcoplasmic reticulum Ca^2+^, quantified with caffeine-induced Ca^2+^ transient amplitude (**E**), or area under the curve (Integral) of the corresponding inward current (I_NCX_) (**F**). **G** Maximum buffer capacity (B_max_, left) and dissociation constant (K_d_, right), determined from buffer curves. **H** Representative total cytosolic Ca^2+^ concentration during I_Ca,L_-triggered Ca^2+^ transients in Ctrl (left) and TnT-R173W (right) iPSC-CM. **I** Total cytosolic Ca^2+^ amplitude. *n* = number of iPSC-CM from 3 to 5 batches. Data are mean ± SEM. *P < 0.05, **P < 0.01 and ***P < 0.001 vs. Ctrl using Student’s *t* test (**B**, **C**, **E**–**G** left, **I**) and the Mann–Whitney *U* test (**G** right)

To further investigate mechanisms underlying impaired Ca^2+^ handling in DCM-TnT-R173W iPSC-CM, epifluorescence was combined with the whole-cell voltage-clamp technique. No significant difference between membrane capacitance of DCM-TnT-R173W and Ctrl iPSC-CM was observed (R173W: 21.65 ± 1.63 pF, n/N = 46/5 vs. Ctrl: 27.38 ± 3.28 pF, n/N = 29/3; Mann–Whitney: *P* = 0.33), indicating comparable cell size. I_Ca,L_ was induced by a voltage-step protocol (0.5 Hz stimulation frequency) and was measured simultaneously with cytosolic Ca^2+^ (Fig. 3A).

The peak amplitude and integral of I_Ca,L_ were both greater in DCM-TnT-R173W, compared to Ctrl. Interestingly, despite greater I_Ca,L_, the triggered Ca^2+^ transient amplitude was smaller in DCM-TnT-R173W compared to Ctrl (Fig. 3C right panel). Diastolic Ca^2+^ levels were comparable in both groups (Fig. 3C left panel).

### Increased intracellular Ca^2+^ buffering in DCM-TnT-R173W iPSC-CM

The Ca^2+^ transient amplitude is determined by various factors such as I_Ca,L_ and SR Ca^2+^ content. Considering increased I_Ca,L_ (Fig. 3B), we subsequently measured SR Ca^2+^ content. After 3–5 min stimulation at 0.5 Hz using the voltage-step protocol described above, myocytes were clamped at − 80 mV and caffeine (10 mM) was applied to induce complete Ca^2+^ release from the SR (Fig. 3D upper panel). The amplitude of the resulting caffeine-induced Ca^2+^ transient was smaller in DCM-TnT-R173W, compared to Ctrl (Fig. 3E).

As the majority of Ca^2+^ released from the SR during caffeine application is extruded out of the cell by the electrogenic Na^+^-Ca^2+^-exchanger (NCX), integration of the resulting NCX current can be used as an index of the “total” amount of Ca^2+^ released from the SR. This was comparable in DCM-TnT-R173W and Ctrl (Fig. 3D middle panel, F), in contrast with the amplitude of the caffeine-induced Ca^2+^ transient, which was smaller in DCM-TnT-R173W. Since the latter is quantified using intracellular Ca^2+^ indicators such as Fluo-3, and intracellular Ca^2+^ buffers such as SR Ca^2+^-ATPase (SERCA) and TnC compete with the indicator for binding to Ca^2+^ ions, the caffeine-induced Ca^2+^ transient represents only the “free” cytosolic Ca^2+^ concentration [59]. Therefore, an increase in intracellular Ca^2+^ buffering may explain the reduced amplitude of the caffeine-induced Ca^2+^ transient, despite comparable measurements of total Ca^2+^.

To quantify intracellular Ca^2+^ buffering, the integral of the caffeine-induced NCX current was plotted against free [Ca^2+^], as determined during the decay of the caffeine-induced Ca^2+^ transient [59, 66]. The data were fitted with a Michaelis–Menten buffer curve (Fig. 3D lower panel):

$$\left[ {{\text{Ca}}^{{2 + }} } \right]_{{{\text{total}}}} = \frac{{B_{{max}} \left[ {Ca^{{2 + }} } \right]_{i} }}{{K_{d} + \left[ {Ca^{{2 + }} } \right]_{i} }}.$$

The maximum buffer capacity *B*_*max*_ was comparable between DCM-TnT-R173W and Ctrl, pointing to a similar amount of cytosolic Ca^2+^-binding sites. In contrast, the dissociation constant *K*_*d*_, which represents the [Ca^2+^]_i_ at which buffers are half saturated, was significantly lower in DCM-TnT-R173W, compared to Ctrl (Fig. 3G). A lower *K*_*d*_ suggests increased affinity of cytosolic Ca^2+^ buffers, thereby resulting in increased Ca^2+^ buffering in DCM-TnT-R173W.

Based on the estimated values of *B*_*max*_ and *K*_*d*_, Ca^2+^-buffer curves were calculated for each individual experiment (Supplementary Fig. 5A), allowing estimation of the time course of changes of total cytosolic Ca^2+^ during I_Ca,L_-triggered Ca^2+^ transient (Fig. 3H). In contrast to the lower free cytosolic Ca^2+^ transient amplitude (Fig. 3C right panel), the amplitude of total Ca^2+^ release in DCM-TnT-R173W was comparable to Ctrl (F﻿ig. 3I) suggesting that apparent alterations in systolic Ca^2+^ transients are mainly due to increased Ca^2+^ buffering.

### Slower decay of free systolic Ca^2+^ transient in TnT-R173W iPSC-CM is due to increased Ca^2+^ buffering

**Fig. 4:**
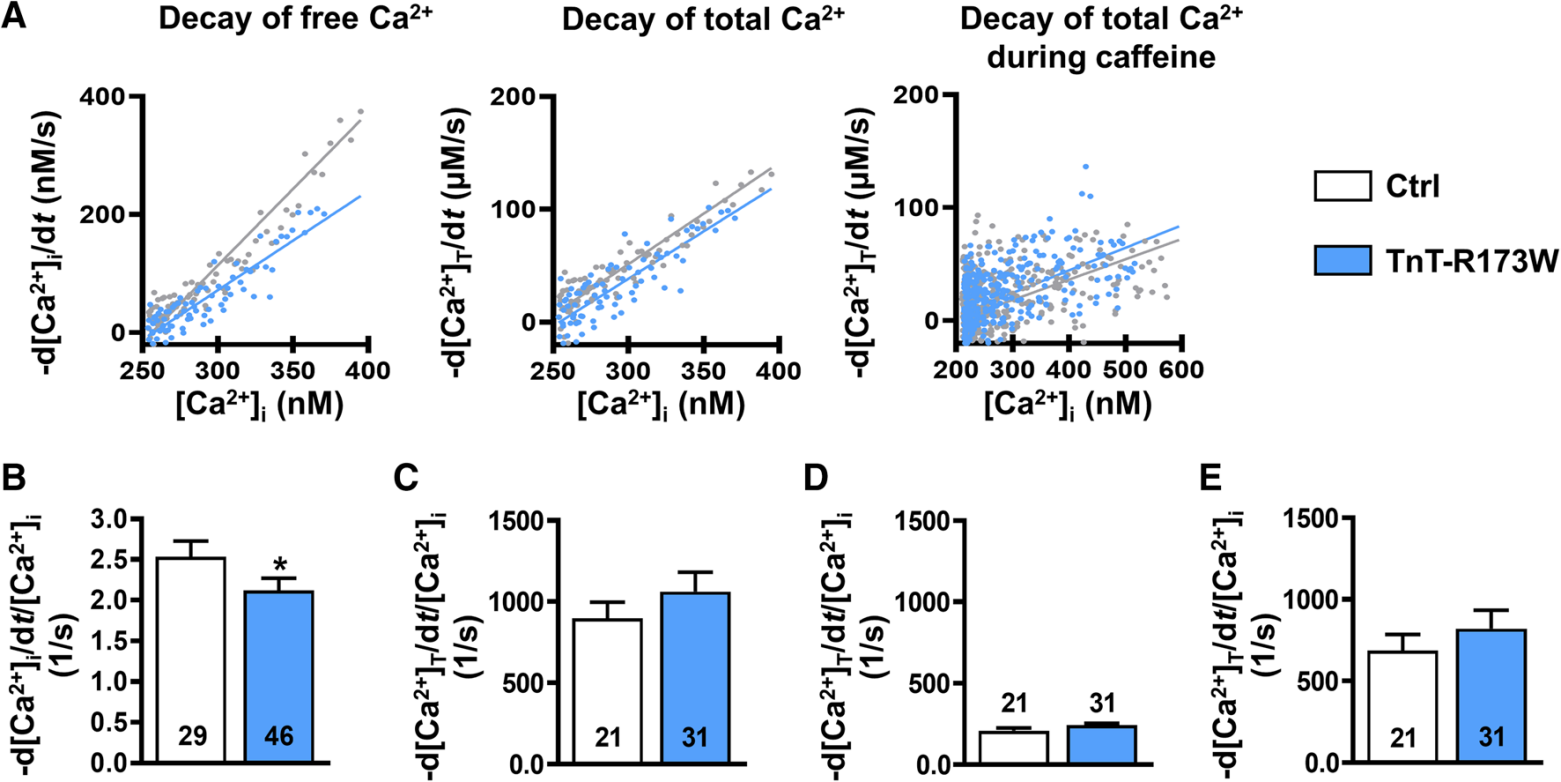
Quantification of decay of free and total Ca^2+^ transient of control (Ctrl) and DCM-TnT-R173W induced pluripotent stem cell-derived cardiomyocytes (iPSC-CM). **A** Representative rate of decay of free Ca^2+^ (-d[Ca^2+^]_i_/d*t*) plotted against free [Ca^2+^]_i_ (left), representative rate of decay of total Ca^2+^ (-d[Ca^2+^]_total_/d*t*) plotted against free [Ca^2+^]_i_ (middle) and representative rate of decay of total Ca^2+^ during caffeine-induced Ca^2+^ transient plotted against the corresponding free [Ca^2+^]_i_ (right) in Ctrl and TnT-R173W iPSC-CM. Slopes are shown as a linear function. **B** Slope of -d[Ca^2+^]_i_/d*t* plotted against [Ca^2+^]_i_. **C** Slope of -d[Ca^2+^]_total_/d*t* plotted against [Ca^2+^]_i_. **D** Slope of -d[Ca^2+^]_total_/d*t* during caffeine plotted against the corresponding [Ca^2+^]_i_. **E** Difference between C and D indicating unaltered [Ca^2+^]_I_ dependence of SERCA-mediated Ca^2+^ removal. *n* = number of iPSC-CM from 3 to 5 batches. Data are mean ± SEM *P < 0.05 vs. Ctrl using Mann–Whitney *U* test (**B**, **C**, **E**) and Student’s *t* test (**D**)

We further assessed whether the measured changes of cytosolic Ca^2+^ buffering can account quantitatively for the observed slowing of the cytosolic free Ca^2+^ transient [10]. Therefore, we plotted the rate of decay of free Ca^2+^ (-d[Ca^2+^]_i_/d*t*) as a function of the free cytosolic Ca^2+^ level (Fig. 4A) and, in accordance with slower decay of the cytosolic Ca^2+^ transient, we found the gradient of this relationship to be smaller in DCM-TnT-R173W (Fig. 4B). In contrast, Fig. 4C shows the rate of decay of total Ca^2+^ (-d[Ca^2+^]_total_/d*t*) plotted against the free [Ca^2+^]_i_ with unaltered slope in DCM-TnT-R173W. The unaltered slope shows that the slowed decay of the systolic free Ca^2+^ transient in DCM-TnT-R173W (﻿Fig. 2B) can be attributed quantitatively to increased Ca^2+^ buffering.

To estimate the contribution of NCX to cytosolic Ca^2+^ removal, we plotted the rate of decay of total Ca^2+^ during caffeine-induced Ca^2+^ transient against the corresponding free cytosolic Ca^2+^ level. The resulting slope was comparable between both groups suggesting unaltered activity of NCX (Fig. 4D). In accordance, the slope of the line relating I_NCX_ to [Ca^2+^]_i_ during decay of caffeine-induced Ca^2+^ transient (Supplementary Fig. 9A, B) showed no difference between groups, confirming unaltered Ca^2+^-dependence of NCX function. Since [Ca^2+^]_i_ dependence of decay rate of total Ca^2+^ (d[Ca^2+^]_total_/d*t*) was unaltered during systolic and caffeine-induced Ca^2+^ transients, it can be concluded that [Ca^2+^]_i_ dependence of SERCA activity is unaltered in DCM-TnT-R173W, which has been estimated based on the difference between the two respective slopes (Fig. 4E).

### Pharmacological increase in myofilament Ca^2+^ affinity reproduces the Ca^2+^ handling phenotype observed in DCM-R173W iPSC-CM

Since TnC is the major cytosolic Ca^2+^ buffer [59], the DCM-TnT-R173W mutation likely results in increased Ca^2+^ affinity of TnC, leading to increased Ca^2+^ buffering and altered Ca^2+^ homeostasis.

**Fig. 5:**
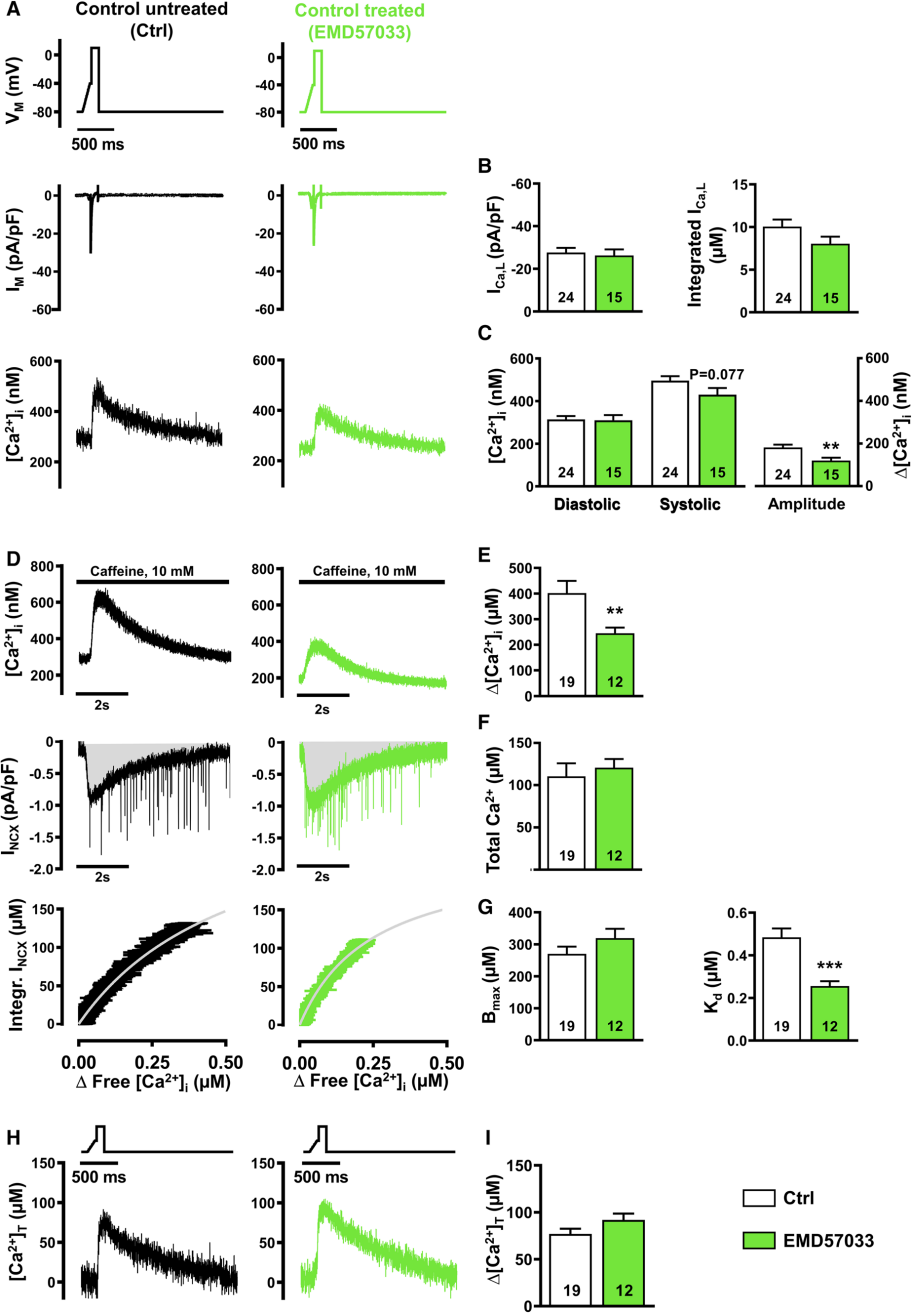
I_Ca,L_ and Ca^2+^ transient (upper), sarcoplasmic reticulum Ca^2+^ load and intracellular Ca^2+^ buffering (middle) and total cytosolic Ca^2+^ concentrations during I_Ca,L_ triggering (lower) in control (Ctrl) induced pluripotent stem cell-derived cardiomyocytes (iPSC-CM) pre-treated with EMD57033 (5 µM). **A** Voltage-clamp protocol (upper), representative simultaneous recordings of I_Ca,L_ (middle) and corresponding I_Ca,L_-triggered Ca^2+^ transients (CaT, lower) in untreated Ctrl iPSC-CM (left) and Ctrl iPSC-CM pre-treated with EMD57033 (right). **B** Peak I_Ca,L_ amplitude (left) and integrated I_Ca,L_ (right). **C** Diastolic and systolic [Ca^2+^]_i_ (left) and Ca^2+^ transient amplitude (right). **D** Representative recordings of caffeine-induced Ca^2+^ transient i.e. free cytosolic Ca^2+^ concentration (upper) with associated depolarising inward current (I_NCX_, middle) in untreated Ctrl iPSC-CM (left) and Ctrl iPSC-CM pre-treated with EMD57033 (right). Integrated I_NCX_ as an index for total cytosolic Ca^2+^ concentration was plotted against corresponding cytosolic free Ca^2+^ concentration (lower). Buffer curves depicting the relationship between cytosolic free and total Ca^2+^ were fitted with hyperbolic functions. **E**, **F** Sarcoplasmic reticulum Ca^2+^, quantified with caffeine-induced Ca^2+^ transient amplitude (**E**), or area under the curve (Integral) of the corresponding inward current (I_NCX_) (**F**). **G** Maximum buffer capacity (B_max_, left) and dissociation constant (K_d_, right), determined from buffer curves. **H** Representative total cytosolic Ca^2+^ concentration during I_Ca,L_-triggered Ca^2+^ transients in untreated Ctrl iPSC-CM (left) and Ctrl iPSC-CM pre-treated with EMD57033 (right). **I** Total cytosolic Ca^2+^ amplitude. *n* = number of iPSC-CM from three batches. Data are mean ± SEM. **P < 0.01 and ***P < 0.001 vs. Ctrl using Student’s *t* test (**B**, **C** left, **F**, **G**, **I**) and the Mann–Whitney *U* test (**C** right, **E**)

**Fig. 6:**
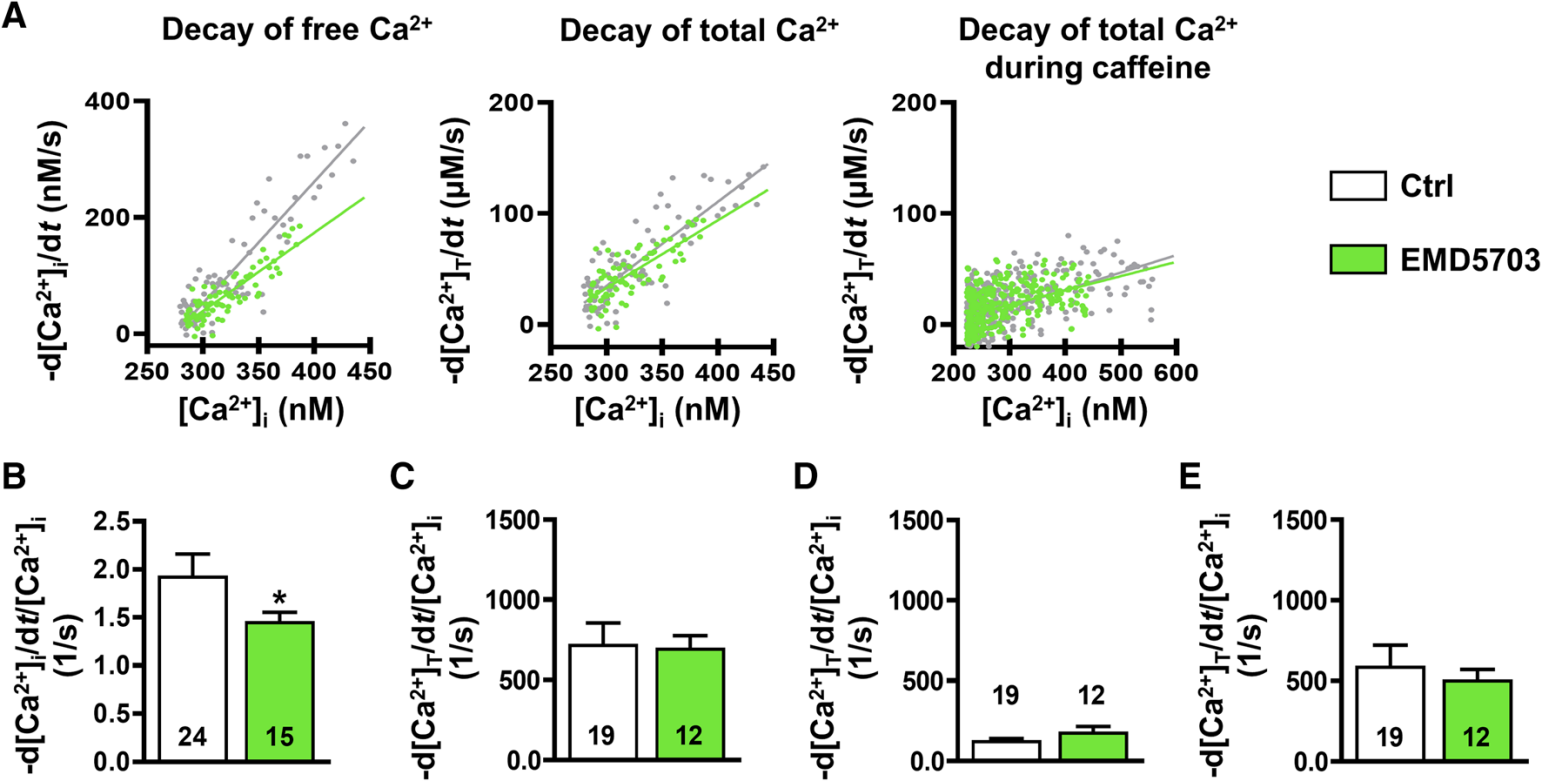
Quantification of decay of free and total Ca^2+^ transient in control (Ctrl) induced pluripotent stem cell-derived cardiomyocytes (iPSC-CM) pre-treated with EMD57033 (5 µM). **A** Representative rate of decay of free Ca^2+^ (-d[Ca^2+^]_i_/d*t*) plotted against free [Ca^2+^]_i_ (left), representative rate of decay of total Ca^2+^ (-d[Ca^2+^]_total_/d*t*) plotted against free [Ca^2+^]_i_ (middle) and representative rate of decay of total Ca^2+^ during caffeine-induced Ca^2+^ transient plotted against the corresponding free [Ca^2+^]_i_ (right) in Ctrl iPSC-CM with and without EMD57033 treatment. Slopes are shown as a linear function. **B** Slope of -d[Ca^2+^]_i_/d*t* plotted against [Ca^2+^]_i_. **C** Slope of -d[Ca^2+^]_total_/d*t* plotted against [Ca^2+^]_I_. **D** Slope of -d[Ca^2+^]_total_/d*t* during caffeine plotted against the corresponding [Ca^2+^]_i_. **E** Difference between C and D indicating unaltered [Ca^2+^]_I_ dependence of SERCA-mediated Ca^2+^ removal. *n* = number of iPSC-CM from three batches. Data are mean ± SEM. *P < 0.05 vs. Ctrl using Mann–Whitney *U* test (**B**–**E**)

To investigate whether increased Ca^2+^ affinity of myofilaments may contribute to Ca^2+^ handling abnormalities observed in DCM-TnT-R173W iPSC-CM, Ctrl iPSC-CM were treated with the Ca^2+^ sensitiser EMD57033 (5 µM, 5 min pre-treatment, Figs. 5, 6) [1]. EMD57033 treatment had no effect on I_Ca,L_ (Fig. 5B). This is in stark contrast to the greater I_Ca,L_ in DCM-TnT-R173W iPSC-CM, an effect which, therefore, appears to be independent of increased Ca^2+^ buffering. Ca^2+^ transient amplitude was, however, smaller in EMD57033-treated iPSC-CM, compared to Ctrl (Fig. 5C right panel).

EMD57033 treatment did not affect SR Ca^2+^ load, as quantified by the integration of the caffeine-induced I_NCX_ (Fig. 5D middle panel, F). In contrast, amplitude of the caffeine-induced Ca^2+^ transient was smaller in EMD57033-treated iPSC-CM, compared to untreated (Fig. 5E), indicating less free [Ca^2+^]_i_ during Ca^2+^ release from the SR. Quantification of buffer properties revealed unaltered total buffer capacity, *B*_*max*_, but a lower dissociation constant, *K*_*d*_, in EMD57033-treated iPSC-CM (Fig. 5G). These data phenocopy the Ca^2+^ handling properties observed in DCM-TnT-R173W iPSC-CM and confirm the effective increase in cytosolic Ca^2+^ buffering in myocytes treated with EMD57033.

The smaller Ca^2+^ transient amplitude in myocytes treated with EMD57033 is unlikely to be caused by the lower total amount of Ca^2+^ released from the SR because the amplitude of the total Ca^2+^ transient calculated based on Ca^2+^ buffer curves (Supplementary Fig. 5C) was unaltered (F﻿ig. 5H, I). Rather, it is more likely due to stronger binding of Ca^2+^ to myofilaments sensitised by EMD57033. The latter also hampers diastolic Ca^2+^ removal from the cytosol since Ca^2+^ must first dissociate from buffers before it can interact with Ca^2+^ removal mechanisms of the SR and the sarcolemma, respectively. Accordingly, decay of cytosolic free Ca^2+^ during I_Ca,L_-triggered SR Ca^2+^ release was slower in EMD57033-treated iPSC-CM (Fig. 6B). In contrast, [Ca^2+^]_i_ dependence of the decay rate of total Ca^2+^ during I_Ca,L_-triggered and caffeine-induced SR Ca^2+^ release was unaltered in EMD57033-treated cells pointing to unchanged SERCA and NCX [Ca^2+^]_i_ dependence (Fig. 6C–E).

**Fig. 7:**
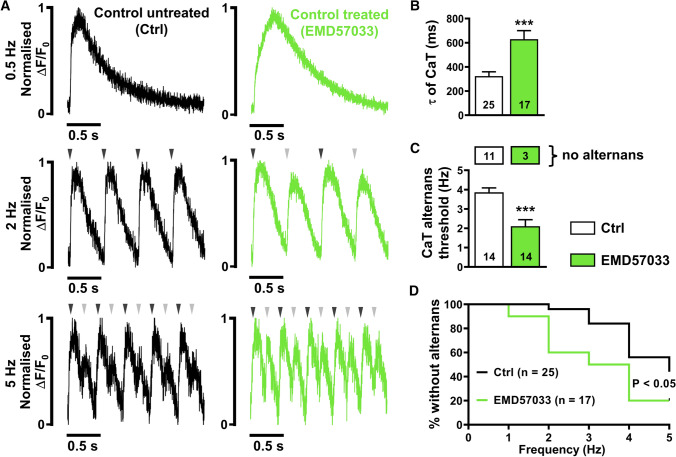
Incidence of Ca^2+^ alternans in control (Ctrl) induced pluripotent stem cell-derived cardiomyocytes (iPSC-CM) pre-treated with EMD57033 (5 µM). **A** Normalised representative traces of Ca^2+^ transients (CaT) at 0.5 Hz (upper), 2 Hz (middle) and 5 Hz (lower) in untreated Ctrl (left) and in Ctrl iPSC-CM pre-treated with EMD57033 (right). Arrowheads indicate electrical stimulation and illustrate when beat-to-beat alterations are present. **B** Ca^2+^ transient time constant of decay (τ). **C** Alternans threshold frequency. Number of myocytes without alternans is shown in boxes above. **D** Kaplan–Meier plot showing the percentage of cells without alternans in relation to the respective pacing. *n* = number of iPSC-CM from 2 to 5 batches. Data are mean ± SEM. *P < 0.05 and ***P < 0.001 vs. Ctrl using the Mann–Whitney *U* test (**B**, **C**), and the Gehan–Breslow–Wilcoxon test (**D**)

To demonstrate that increased Ca^2+^ buffering is sufficient to increase alternans susceptibility, field stimulation experiments were performed in EMD57033-treated iPSC-CM (Fig. 7). Similar to DCM-TnT-R173W, Ca^2+^ transient upstroke was slower (Supplementary Fig. 4B) and time constant of decay was greater in EMD57033-treated iPSC-CM, compared to Ctrl (Fig. 7B). Furthermore, EMD57033-treated iPSC-CM demonstrated higher susceptibility for Ca^2+^ alternans, as shown by the Kaplan–Meier curve (Fig. 7D), and a lower threshold frequency for Ca^2+^ transient alternans, compared to Ctrl (Fig. 7C).

### Blebbistatin reduces alternans susceptibility in DCM-TnT-R173W iPSC-CM

**Fig. 8:**
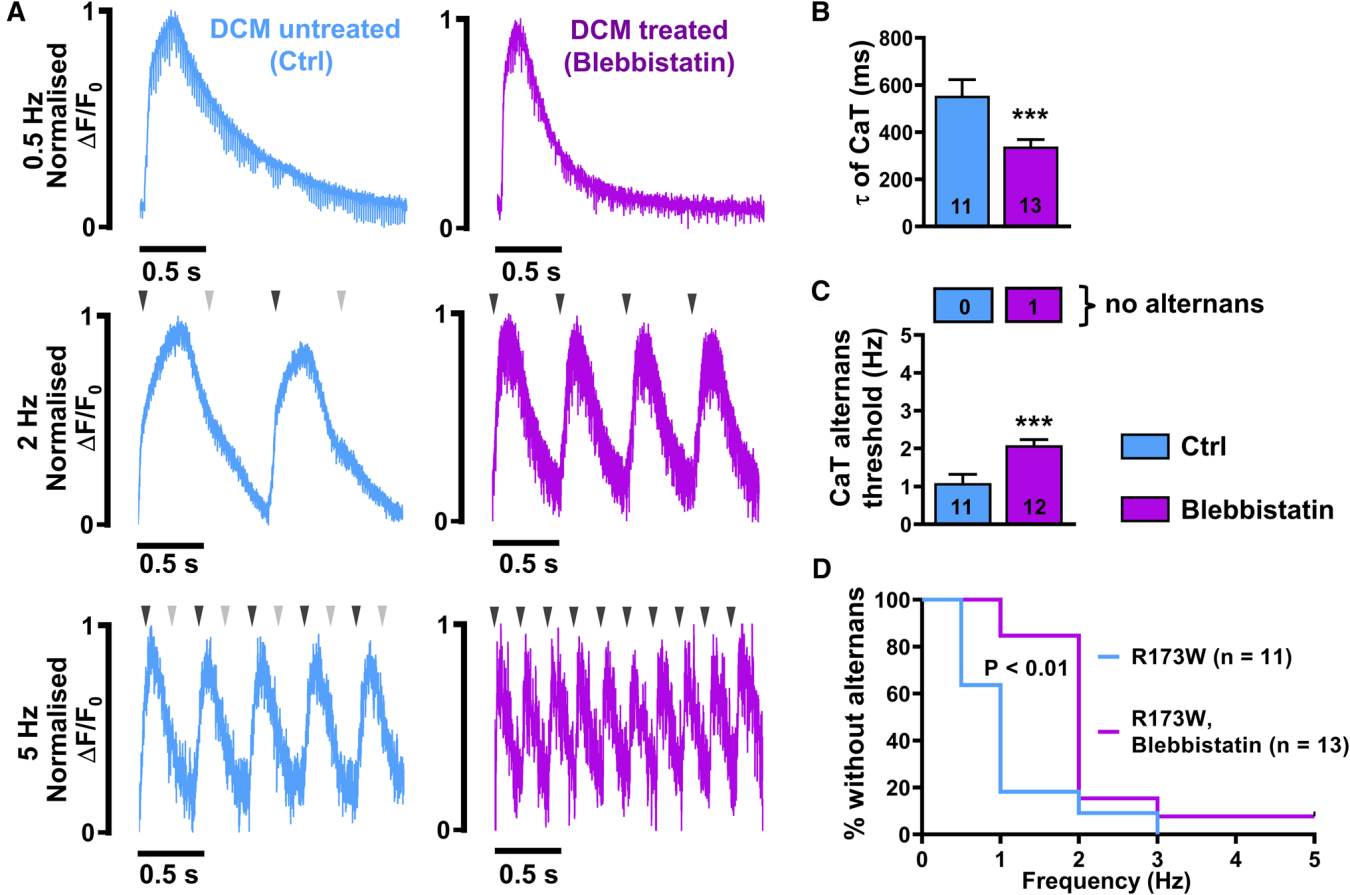
Incidence of Ca^2+^ alternans in DCM-TnT-R173W induced pluripotent stem cell-derived cardiomyocytes (iPSC-CM) pre-treated with blebbistatin (10 µM). **A** Normalised representative traces of Ca^2+^ transients (CaT) at 0.5 Hz (upper), 2 Hz (middle) and 5 Hz (lower) in untreated TnT-R173W iPSC-CM (left) and in TnT-R173W iPSC-CM pre-treated with blebbistatin (right). Arrowheads indicate electrical stimulation and illustrate when beat-to-beat alterations are present. **B** Ca^2+^ transient time constant of decay (τ). **C** Alternans threshold frequency. Number of myocytes without alternans is shown in boxes above. **D** Kaplan–Meier plot showing the percentage of cells without alternans in relation to the respective pacing. *n* = number of iPSC-CM from two batches. Data are mean ± SEM. **P < 0.01 vs. Ctrl using the Mann–Whitney *U* test (**B**, **C**), and the Gehan–Breslow–Wilcoxon test (**D**)

Blebbistatin is an inhibitor of myosin ATPase and has been suggested to reduce Ca^2+^ affinity of myofilaments [1]. To test whether the proarrhythmic phenotype of DCM-TnT-R173W iPSC-CM may be rescued by normalisation of myofilament Ca^2+^ affinity, Ca^2+^ transients were recorded in electrically field stimulated DCM-TnT-R173W iPSC-CM treated with blebbistatin (10 µM, 20 min pre-treatment, Fig. 8). As shown in Fig. 8B, blebbistatin treatment normalised Ca^2+^ transient time constant of decay. In addition, blebbistatin reduced susceptibility to Ca^2+^ transient alternans in DCM-TnT-R173W iPSC-CM and increased the threshold frequency at which Ca^2+^ transient alternans occurred (Fig. 8C, D).

## Discussion

In the current study, iPSC-CM with the DCM-TnT-R173W mutation were used to assess Ca^2+^ handling abnormalities and examine the arrhythmogenic propensity of patients with DCM. Single-cell patch-clamp experiments revealed increased intracellular Ca^2+^ buffering in DCM-TnT-R173W iPSC-CM. In addition, it could be demonstrated that these alterations in cytosolic Ca^2+^ handling contribute to AP and Ca^2+^ transient alternans as a potential underlying substrate for increased arrhythmogenesis in DCM patients harbouring the DCM-TnT-R173W mutation. Of note, treatment with blebbistatin, a myosin ATPase inhibitor causing decreased myofilament Ca^2+^ sensitivity [1], reduced the occurrence of Ca^2+^ alternans in DCM-TnT-R173W iPSC-CM. Our findings suggest that modulation of myofilament Ca^2+^ sensitivity may represent a potential anti-arrhythmic concept in DCM patients, in particular in those harbouring mutations in TnT.

### Cardiac arrhythmia mechanisms in DCM patients

DCM patients are more prone to cardiac arrhythmia development, with the main reasons for mortality being end-organ dysfunction due to heart failure or arrhythmia-related death [65]. Premature ventricular contractions and non-sustained ventricular tachycardia are common in DCM and are observed in up to 90% and 60% of patients, respectively. Cardiac arrest can occur due to monomorphic or polymorphic ventricular tachycardia, degenerating to ventricular fibrillation [27].

A variety of mechanisms have been proposed to contribute to arrhythmogenesis in patients with DCM, but the primary cause is not well understood [27]. DCM patients often present with multiple patchy areas of replacement fibrosis, which can act as sites for re-entry, one of the most common mechanisms underlying ventricular tachycardia and sudden cardiac death [40, 55]. Other hypotheses focus on abnormal wall stretch, causing alterations in ventricular refractoriness and predisposing the patient to abnormal automaticity and triggered activity [6].

Given the broad spectrum of genetic and non-genetic contributors to DCM pathophysiology, the identification of a common pathomechanism underlying arrhythmogenesis in all DCM patients is almost impossible. Valvular heart disease, excessive alcohol consumption, hypertension and infectious diseases, for example, are accepted etiological factors associated with disease-specific remodelling pathways leading to DCM [42]. Nevertheless, in about 40% of patients with DCM, underlying genetic factors are thought to play a role [18]. Most mutations causing DCM are located in genes encoding for cytoskeletal, sarcolemmal and sarcomeric proteins [31, 42]. A “disruption” in the link between these three components and consecutive disturbance of ion-channel function have been proposed as the “final common pathway” in DCM arrhythmogenesis [65].

Many DCM-causing mutations have also been shown to affect multiple aspects of Ca^2+^ homeostasis in cardiac myocytes, including altered binding of Ca^2+^ to myofilaments, as well as disrupted expression of Ca^2+^ handling proteins. It follows, therefore, that abnormal Ca^2+^ handling may also play a potentially key role in DCM-related arrhythmogenesis [30, 64].

### Altered cytosolic Ca^2+^ handling in DCM patients

Ca^2+^ is a major mediator of excitation–contraction coupling [4] and specific alterations in cellular Ca^2+^ handling are likely to contribute to impaired contractile function in patients with DCM [31]. Accordingly, decreased amplitude of systolic Ca^2+^ transients appears to be a common finding in all DCM models in which cytosolic Ca^2+^ handling has been investigated [2, 36, 37, 62, 63]. Reduced Ca^2+^ transient amplitude has been suggested to result from reduced SR Ca^2+^ content, which may be due to increased diastolic Ca^2+^ leak from the SR mediated by leaky type 2 ryanodine receptor channels (RyR2) [2]. Reduced SR Ca^2+^ content may also result from slower Ca^2+^ reuptake into the SR due to reduced activity of SERCA [43].

Here, we describe another mechanism which could also contribute to reduced Ca^2+^ transient amplitude in DCM patients. Our experiments suggest that reduced Ca^2+^ transient amplitude in DCM-TnT-R173W cardiomyocytes [63] results from increased Ca^2+^ buffering due to increased binding of Ca^2+^ to myofilaments [14]. This is consistent with previous publications showing that the DCM-TnT-R173W mutation limits binding of protein kinase A to local sarcomere microdomains, thereby attenuating phosphorylation of TnI [9]. The latter might contribute to increasing the Ca^2+^ sensitivity of TnC and increase Ca^2+^-myofilament binding within the physiologically relevant range (Supplementary Figs. 5B, D).

It is important to note that contractile function is attenuated in DCM-TnT-R173W myocytes and engineered heart muscle constructs (EHM) [9, 39]. A contributing factor might be the reduced interaction of TnT with tropomyosin in the presence of the DCM-TnT-R173W mutation, which is located within one of the two tropomyosin binding regions of TnT [9, 24]. This may affect correct relocation of tropomyosin following Ca^2+^ binding to TnC as well as freeing of the myosin-binding sites on actin, thereby limiting contraction. A similar discrepancy has been shown in skinned muscle fibres in response to caffeine, which sensitises the force response to lower Ca^2+^ concentrations without affecting Ca^2+^ binding to TnC [53]. Taken together it is important to consider alterations in the “apparent” Ca^2+^ affinity of contractile proteins determined by analysis of contractile function in response to variations of [Ca^2+^]_i_ separately from alterations in the Ca^2+^ binding affinity of myofilaments. The latter represent the major Ca^2+^ buffers in cardiac myocytes and alterations in binding affinity are thought to have relevant impact on cellular Ca^2+^ homeostasis [59].

### Increased susceptibility to arrhythmogenic AP and Ca^2+^ transient alternans in DCM patients

Increased Ca^2+^ buffering has a major impact on Ca^2+^ handling and arrhythmogenesis [14]; Ca^2+^ influences cellular electrophysiology via the modulation of Ca^2+^-dependent ion channels and transporters in the sarcolemma, such as the L-type Ca^2+^ channel and NCX. It, therefore, follows that altered Ca^2+^ handling may also contribute to arrhythmogenesis in patients with DCM. Surprisingly little is known about the role abnormal Ca^2+^ handling plays in arrhythmogenesis in patients with DCM. Previous publications have shown that increased incidence of spontaneous Ca^2+^ release events from the SR during diastole may contribute to arrhythmogenesis, particularly in patients with Duchenne Muscular Dystrophy (DMD)-associated cardiomyopathy [2]. The released Ca^2+^ is extruded from the myocyte by NCX, which brings 3 Na^+^ ions per extruded Ca^2+^ ion into the cell, giving rise to a depolarising inward current. If this current is large enough, it will trigger a new action potential and ectopic activity, with the potential to initiate cardiac arrhythmias [20, 70, 71].

Here, we demonstrate for the first time that cardiomyocytes from patients harbouring a DCM-causing mutation are prone to developing arrhythmogenic AP and Ca^2+^ transient alternans. The maximum slope of the AP restitution curves did not exceed one (Fig. 1B), which points towards Ca^2+^-driven alternans as opposed to alternans based on AP which requires a steeper restitution slope [15, 73]. In addition, the alterations in Ca^2+^ handling properties seen in DCM-TnT-R173W iPSC-CM could be reproduced in control iPSC-CM treated with the Ca^2+^ sensitiser EMD57033. This highlights increased Ca^2+^ buffering as a major contributor to impaired Ca^2+^ handling and increased susceptibility to arrhythmogenic alternans in DCM-TnT-R173W iPSC-CM.

Ca^2+^ alternans is enhanced by factors which increase SR Ca^2+^ release and reduce Ca^2+^ sequestration from the cytosol [73]. Increased Ca^2+^ buffering has previously been shown to reduce Ca^2+^ reuptake into the SR [10, 59]. In the present study, Ca^2+^ transient decay was slower in DCM-TnT-R173W iPSC-CM. We suggest that this is predominantly due to slowed SERCA and NCX-mediated Ca^2+^ removal from the cytosol secondary to increased Ca^2+^ buffering by myofilaments. Since NCX-mediated Ca^2+^ removal is electrogenic, the slower Ca^2+^ transient decay, it generates a depolarising inward current resulting in slower repolarisation of the membrane potential during diastole. Indeed, further analysis of diastolic potentials during optical AP recordings revealed a significantly increased diastolic potential preceding every even (“pathological”) beat compared with every odd (“physiological”) AP. This is consistent with incomplete diastolic extrusion of intracellular Ca^2+^ in DCM-TnT-R173W iPSC-CM and persistence of NCX current (Supplementary Fig. 3F, G). The higher diastolic membrane potential prevents recovery from inactivation of voltage-gated ion channels thereby leading to impaired AP upstroke and/or duration. Since the abnormal AP appears not sufficient for triggering full SR Ca^2+^ release, cytosolic Ca^2+^ is reduced to its initial state without remaining NCX current, thereby allowing full repolarization of the membrane potential and the alternans cycle starts all over again [73].

AP and Ca^2+^ transient alternans may lead to spatial electrical heterogeneity, providing a substrate for arrhythmogenic activity [16, 46]. Interestingly, a similar arrhythmogenic mechanism has been proposed by Baudenbacher et al*.* in mouse models harbouring TnT mutations causing hypertrophic cardiomyopathy [1]. The authors demonstrated that the risk of developing ventricular tachycardia was directly proportional to the degree of Ca^2+^ sensitisation caused by the mutation. Furthermore, in vitro studies demonstrate that HCM-causing mutations sensitising myofilaments to Ca^2+^ are associated with high risk of sudden cardiac death [17, 20, 26, 57, 72]. There is also evidence for increased myofilament Ca^2+^ sensitivity in ventricular myocytes after myocardial infarction and also from patients with heart failure. Both diseases are associated with a high incidence of ventricular tachycardia and sudden cardiac death [68, 69, 74]. Our data show, for the first time, that increased Ca^2+^ buffering and increased susceptibility to AP and Ca^2+^ alternans also occur in myocytes from DCM-TnT-R173W patients. The extent to which our findings are valid in other subsets of DCM patients requires further investigation.

### Potential limitations

In the present study, we used iPSC-CM from DCM patients harbouring the TnT-R173 mutation. iPSC-CM represent myocytes at an immature developmental stage. iPSC-CM exhibit poor co-localisation between I_Ca,L_ channels and RYR2 [54], resulting in more internal, non-coupled RyRs being activated by the subsequent rise in [Ca^2+^]_i_ as opposed to direct activation by I_Ca,L_ channels [32, 78]. Nevertheless, iPSC-CM resemble adult ventricular cardiomyocytes in many aspects of cellular electrophysiology, Ca^2+^ handling and contractile function [12, 23]. In addition, human iPSC-CM present a readily available human model of cardiac myocytes which can be generated on demand in large quantities [7, 11, 13], making them a promising model to investigate electrophysiological abnormalities in patients with inherited cardiac arrhythmias [22, 44, 77].

Our data show significant upregulation of I_Ca,L_ in DCM-TnT-R173W iPSC-CM. In contrast, SR Ca^2+^ content was unchanged in DCM-TnT-R173W iPSC-CM (Fig. 3F). Since the latter is mainly determined by the Ca^2+^ influx-efflux balance, this points to increased diastolic Ca^2+^ efflux [47, 67]. Accordingly, the amount of Ca^2+^ removed by NCX correlated with the Ca^2+^ influx mediated by I_Ca,L_ (Supplementary Fig. 9D, E). In addition, Ca^2+^ removal by forward mode NCX was increased in DCM-TnT-R173W iPSC-CM to compensate for the higher Ca^2+^ influx through upregulated I_Ca,L_. The mechanisms underlying increased I_Ca,L_ are beyond the scope of the present study. Nevertheless, unaltered mRNA expression of the underlying Ca^2+^ channel subunit, Cav1.2, renders intrinsic differences of its expression levels between DCM-TnT-R173W and Ctrl iPSC-CM unlikely (Supplementary Fig. 6). Further analysis of the biphasic I_Ca,L_ inactivation revealed unaltered time course of fast I_Ca,L_ decay (τ_fast_, Supplementary Fig. 7A left panel), which is thought to be mainly due to Ca^2+^ dependent inactivation of I_Ca,L_ [3]. We, therefore, conclude that increased I_Ca,L_ in DCM-TnT-R173W is unlikely due to reduced Ca^2+^-dependent inhibition of I_Ca,L_ in response to reduced free cytosolic Ca^2+^ levels. In accordance, increased cytosolic buffering due to EMD57033 leads to a similar decrease in Ca^2+^ transient amplitude but had no effect on I_Ca,L_. I_Ca,L_ is regulated by various post-transcriptional and post-translational mechanisms including miRNA-dependent inhibition, phosphorylation and expression of accessory units [19, 56]. Future studies are necessary to investigate whether these mechanisms contribute to I_Ca,L_ alterations in DCM patients in general, but also those harbouring the TnT-R173W mutation.

Despite greater I_Ca,L_, the triggered Ca^2+^ transient amplitude was smaller in DCM-TnT-R173W compared to Ctrl. Based on our experiments, we conclude that this is largely due to increased cytosolic Ca^2+^ buffering. In accordance, the total amount of Ca^2+^ released from the SR during I_Ca,L_-triggered Ca^2+^ transients was unaltered in DCM-TnT-R173W (Fig. 3H). Nevertheless, coupling efficiency between Ca^2+^ influx and total Ca^2+^ release was reduced in DCM-TnT-R173W (Supplementary Fig. 8A). Therefore, impaired interaction between L-type Ca^2+^ channel and RyR2 may additionally contribute to reduced free I_Ca,L_-triggered Ca^2+^ transient amplitude.

To quantify cytosolic Ca^2+^ buffering, we employed a method that allows investigation of Ca^2+^ homeostasis in intact cardiomyocytes [66]. Intracellular Ca^2+^ has been quantified by the fluorescent Ca^2+^ indicator Fluo-3, which represents an intracellular Ca^2+^ buffer itself. We assume that the contribution of Fluo-3 to intracellular Ca^2+^ buffering is comparable between experimental groups and does not contribute to the differences observed in the present study. Based on the dissociation constant (*K*_*d*_ = 0.864 µM) and an estimated buffer concentration (b_max_) of 100 µM, the contribution of Fluo-3 to the calculated buffer curves is illustrated in Supplementary Fig. 5. In addition, the employed techniques do only allow indirect conclusions on altered Ca^2+^-binding to the troponin complex. Direct quantification of Ca^2+^ binding to troponin C will require further biochemical analysis that are beyond the scope of the present study [61].

In the present study, we investigated Ca^2+^ handling abnormalities in iPSC-CM carrying a specific mutation in cardiac TnT that has been associated with the occurrence of DCM. Given the multifactorial aetiology of DCM, it is unclear whether reduced Ca^2+^ uptake by SERCA, secondary to increased Ca^2+^ buffering by myofilaments may represent a “final common pathway” underlying arrhythmogenesis in DCM patients [65]. Nevertheless, early studies in ventricular biopsies from DCM patients also revealed a decreased rate of diastolic Ca^2+^ reuptake into the SR [36]. Furthermore, increased Ca^2+^ sensitivity of contraction has also been found in patients and a dog model of pacing induced DCM [45, 75]. The differences in Ca^2+^ sensitivity were abrogated after treatment with the catalytic subunit of PKA, suggesting that, as in DCM caused by TnT-R173W mutations, the increased Ca^2+^ sensitivity of myofilaments may be due to a reduction in PKA-mediated phosphorylation of myofibrillar regulatory proteins.

### Outlook

Based on our findings and given the fact that increased myofilament affinity for Ca^2+^ may contribute to arrhythmogenesis in various cardiac diseases, modulation of myofilament Ca^2+^ sensitivity may represent an important novel concept to prevent cardiac arrhythmias [1, 59].

Targeting Ca^2+^ binding of myofilaments is a classical therapeutic concept to improve contractile dysfunction in heart failure patients. Levosimendan and omecamtiv represent traditional drugs aiming to improve contractile force by increasing Ca^2+^ sensitivity and Ca^2+^-myofilament binding [38, 49]. Nevertheless, it is important to note that levosimendan also increases the incidence of ventricular arrhythmias in patients with heart failure, likely due to alternans of Ca^2+^ [1, 20].

Blebbistatin is an inhibitor of the myosin ATPase and has been shown to prevent the occurrence of Ca^2+^ alternans in mouse hearts harbouring Ca^2+^-sensitising TnT mutations in vitro [1]. According to our data, blebbistatin also prevents Ca^2+^ alternans in DCM-TnT-R173W iPSC-CM. Similar to blebbistatin, mavacamten, a small molecule modulator of β-cardiac myosin, which has been recently evaluated in patients with hypertrophic cardiomyopathy, has also been shown to reduce Ca^2+^ affinity of myofilaments [48]. Both blebbistatin and mavacamten may, therefore, represent interesting lead compounds for the development of novel anti-arrhythmic concepts.

## Supplementary Information

Below is the link to the electronic supplementary material.

Supplementary file1 (DOCX 2314 KB)

## Data Availability

All available data are incorporated into this article and its online supplementary material.
