# Supplementary material for: Increased cytosolic calcium buffering contributes to a cellular arrhythmogenic substrate in iPSC-cardiomyocytes from patients with dilated cardiomyopathy
Source: Basic Res Cardiol. 2022 May 2;117(1):5. doi: 10.1007/s00395-022-00912-z (PMC9061684; doi:10.1007/s00395-022-00912-z)
Supplement: Supplementary file 1 — Supplementary file1 (DOCX 2314 KB) [file 395_2022_912_MOESM1_ESM.docx]

**SUPPLEMENTAL MATERIALS**

**Increased cytosolic calcium buffering contributes to a cellular arrhythmogenic substrate in iPSC-cardiomyocytes from patients with dilated cardiomyopathy**

Philipp Jung^1,2^*, Fitzwilliam Seibertz, MSc^1,2^*, Funsho E. Fakuade, PhD^1,2^, Nadezda Ignatyeva, MSc^2,3^, Shrivatsan Sampathkumar, MSc^2,3^, Melanie Ritter^1,2^, Housen Li, PhD^4,5^, Fleur E. Mason, PhD^1,2^, Antje Ebert, PhD^2,3#^, Niels Voigt, MD^1,2,5#^

^1^Institute of Pharmacology and Toxicology, University Medical Center Göttingen, Göttingen, Germany

^2^DZHK (German Center for Cardiovascular Research), Partner Site Göttingen, Germany

^3^Heart Research Center, Department of Cardiology and Pneumology, University Medical Center Göttingen, Göttingen, Germany

^4^ Institute for Mathematical Stochastics, Georg-August University Göttingen, Göttingen

^5^Cluster of Excellence "Multiscale Bioimaging: from Molecular Machines to Networks of Excitable Cells" (MBExC), University of Göttingen, Göttingen, Germany

**Running title:** Increased Ca^2+^ buffering in DCM-TnT-R173W iPSC-CM

*The first two authors contributed equally to this study.

**^#^Corresponding Authors:**

Niels Voigt, Institute of Pharmacology and Toxicology, Robert-Koch-Straße 40, 37075 Göttingen, Germany, Tel.: 00495513965174, Fax: 00495513965169,
E‑mail: [niels.voigt@med.uni-goettingen.de](mailto:niels.voigt@med.uni-goettingen.de)
ORCID ID: 0000-0001-8230-2341

Antje Ebert, Department of Cardiology and Pneumology, Robert-Koch-Straße 40, 37075 Göttingen, Germany. Tel.: 00495513966309,
E-mail: [antje.ebert@med.uni-goettingen.de](mailto:antje.ebert@med.uni-goettingen.de)
ORCID ID: 0000-0002-3642-242X

**Supplemental Methods**

**Culture and maintenance of human iPSC.** The human iPSC lines used in this study have previously been published [5, 7, 8]. Human iPSC were grown on matrigel-coated plates (VWR), as previously described [1, 3], in chemically defined E8 medium [1] which was changed daily. Cells were passaged every 4 days using EDTA (Life Technologies). Healthy control and DCM iPSC as published in [5, 7] were a kind gift from Joseph C. Wu (Stanford University, CA).

**Pluripotency and cardiac marker analysis.** Human iPSC or iPSC-CM were grown on matrigel-coverslips. For human iPSC, immunofluorescence staining was performed for Oct3/4 (POU5F1, R&D Systems) and Tra-1-81 (PODXL, Stemgent) as previously described [2, 3, 7]. DAPI was used for detection of nuclei. Human iPSC-CM were immunostained with antibodies against cardiac troponin T (Abcam) and sarcomeric alpha-actinin (Sigma). Coverslips were mounted on glass slides using Fluromount G (SouthernBiotech). Images were acquired with a plan apochromat (63x/1.40 oil DIC M27) objective using an inverted confocal microscope (LSM 710 Meta, Zeiss) and ZEN software (Zeiss).

**Analysis of mRNA expression levels.** Total RNA was isolated from human iPSC-CM using a RNeasy Kit (Qiagen) as per the manufacturer’s instructions. RNA was reverse-transcribed into cDNA using a High-Capacity cDNA Reverse Transcription Kit with RNase Inhibitor (Life Technologies). Quantitative real-time PCR (qRTPCR) was performed using Reverse Transcription Supermix (BioRad) and the Hs00167681_m1 as well as Hs03003631_g1 TaqMan probes (Thermo Fisher Scientific), utilising Real-Time PCR Detection System (Life Technologies).

**Supplemental Figures**

**
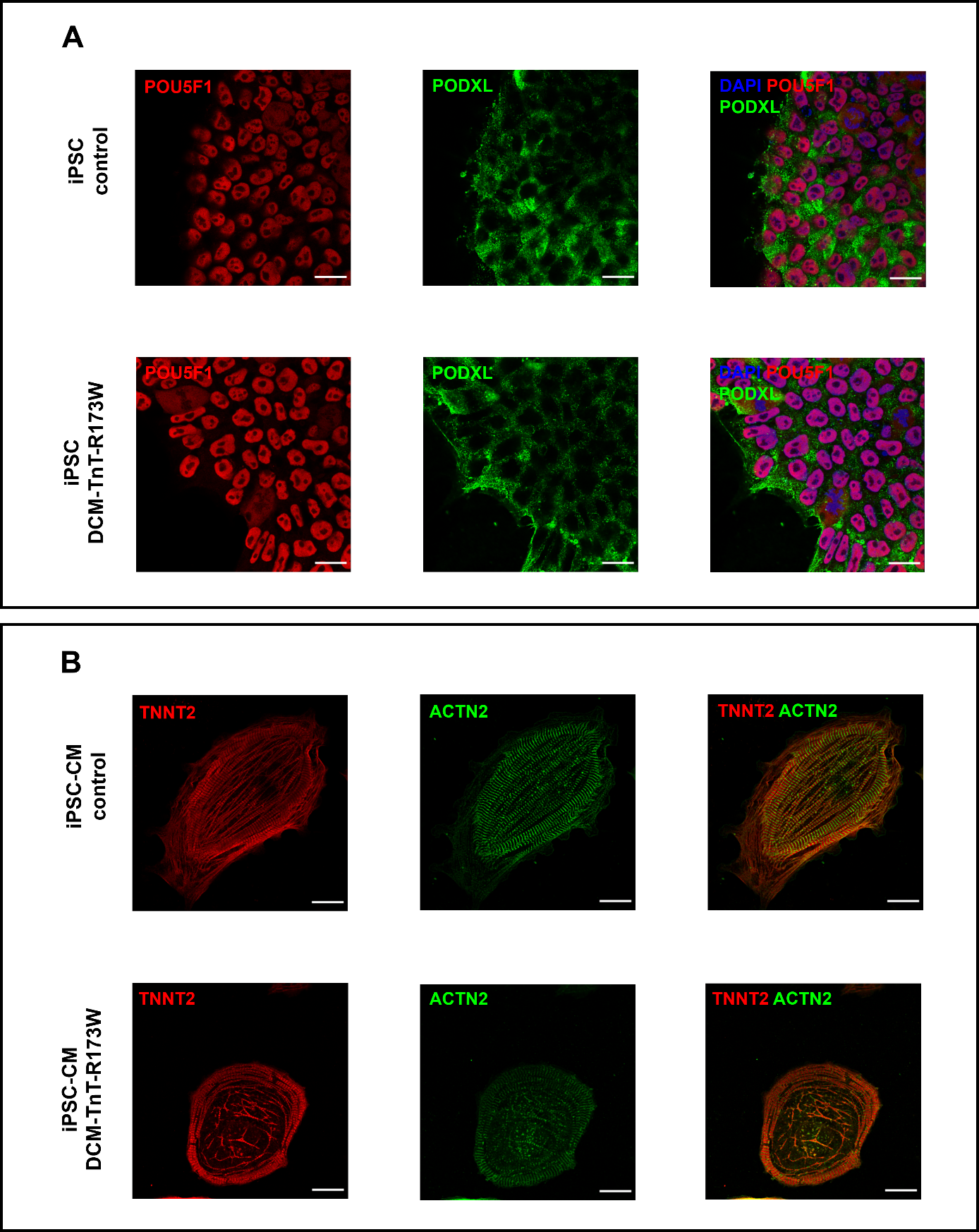
**

**Supplemental Figure 1:** **Control and DCM-TnT-R173W induced pluripotent stem cells (iPSC) express comparable levels of pluripotency markers. Following cardiac differentiation, iPSC-CM express regular levels of cardiac markers.
A**, Immunohistochemistry and confocal imaging for pluripotency markers markers (Oct3/4 [POU5F1], and Tra-1-81 [PODXL]) in control (Ctrl) and DCM patient-derived (DCM-TnT-R173W) iPSC. Scale bar, 20 μm. **B**, Immunohistochemistry and confocal imaging of cardiac markers (cTnT [TNNT2] and SAA [ACTN2]) in Ctrl and DCM-TnT-R173W iPSC-CM. Scale bar, 20 μm.

**
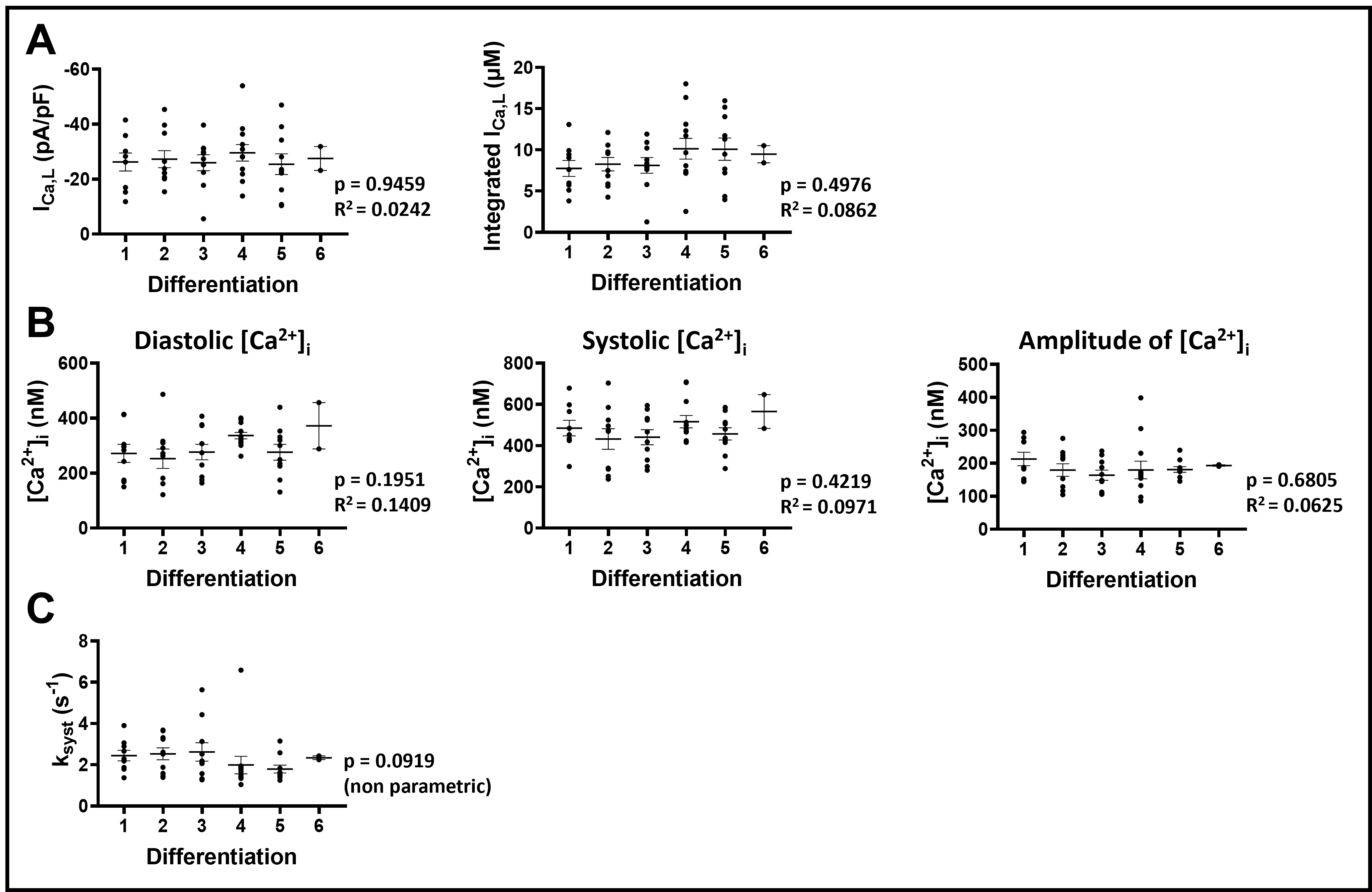
**

**Supplemental Figure 2: Negligible clustering of experimental data in 6 different differentiations of wild type induced pluripotent stem-cell derived cardiomyocytes (iPSC-CM).
A,** Peak I_Ca,L_ amplitude (left) and integrated I_Ca,L_ (right). **B,** Diastolic [Ca^2+^]_i_ (left), systolic [Ca^2+^]_i_ (middle) and Ca^2+^ transient amplitude (right). **C,** Constant of Ca^2+^ transient decay. Individual values and scatter plots are shown, which indicate mean±SEM. P value and R^2^ are shown beside each plot as determined by one-way ANOVA (**A, B**), or Kruskal-Wallis test for non-parametric data sets (**C**).

**
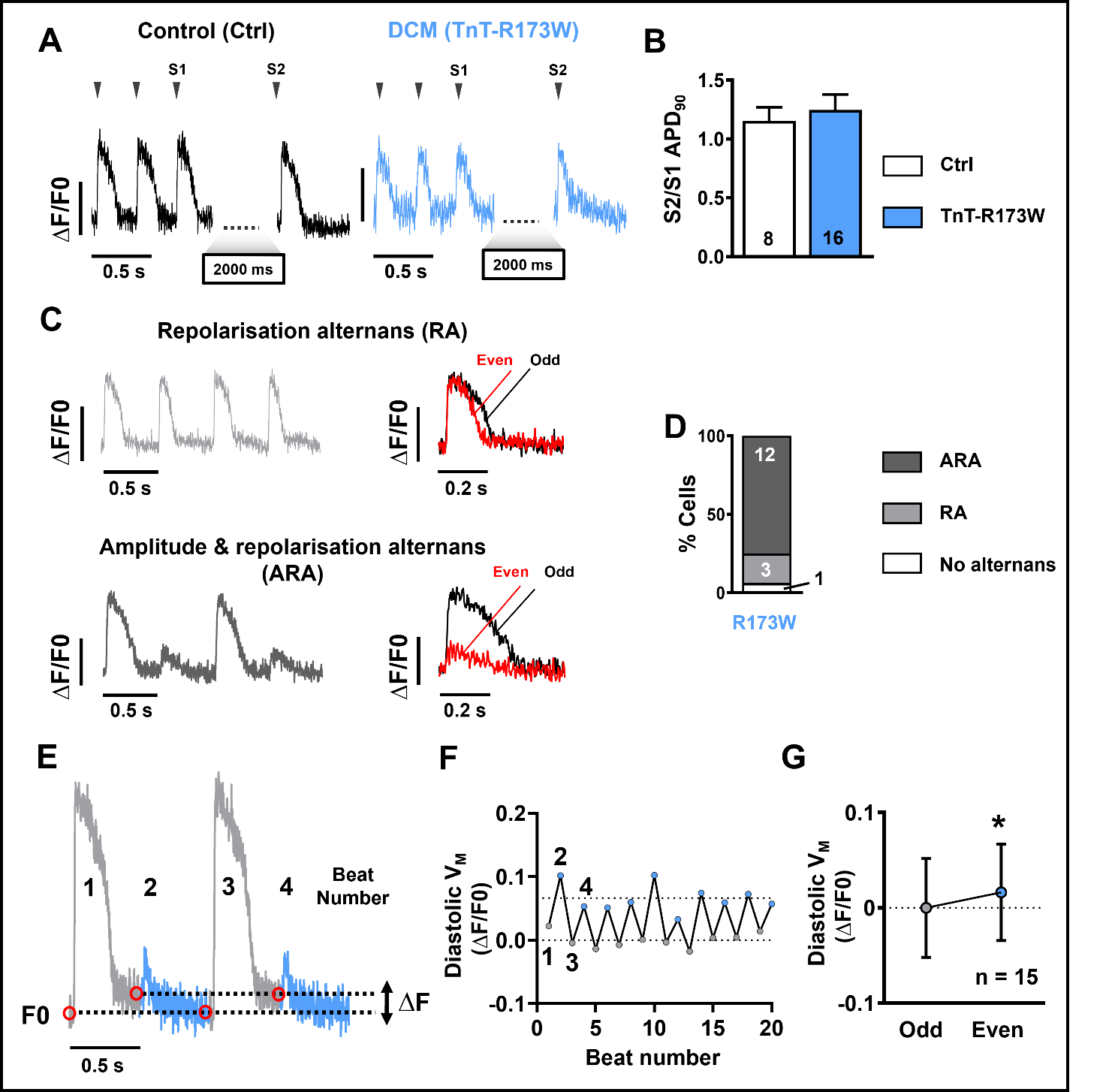
**

**Supplementary Figure 3:** **Optical action potential (AP) characteristics in control (Ctrl) and DCM-TnT-R173W induced pluripotent stem-cell derived cardiomyocytes (iPSC-CM).
A**, Normalised representative traces of optical AP post-rest potentiation in Ctrl (left) and TnT-R173W (right) iPSC-CM. 3 Hz stimulation (S1) was paused for 2 s prior to a post-pause extrastimulus (S2). **B**, Relative post-pause AP duration at 90% repolarisation (APD_90,_ S2) compared with the pre-pause APD_90_ (S1). **C**, Types of AP alternans observed in TnT-R173W iPSC-CM as quantified following analysis with a discrete Fourier transform spectral method [4, 6]. **D,** Percentage of variation in alternans characteristics in all TnT-R173W iPSC-CM optical AP traces. **E**, Representative trace of TnT-R173W iPSC-CM optical AP and resting membrane potential (V_M_) alternans during 2 Hz stimulation. The blue traces indicate the even ‘pathological’ beats. F0 indicates the optical take off potential of every odd ‘physiological’ beat which is increased for every even beat (ΔF). **F**, Representative example of beat-to-beat changes in optical diastolic V_M_ in TnT-R173W iPSC-CM over 20 consecutive beats. **G**, Quantification of diastolic V_M_ changes for all averaged odd and even beats in TnT-R173W iPSC-CM. n = number of iPSC-CM from 2-3 batches. Data are mean±SEM. *P < 0.05 vs. odd using Student’s t-test.

^
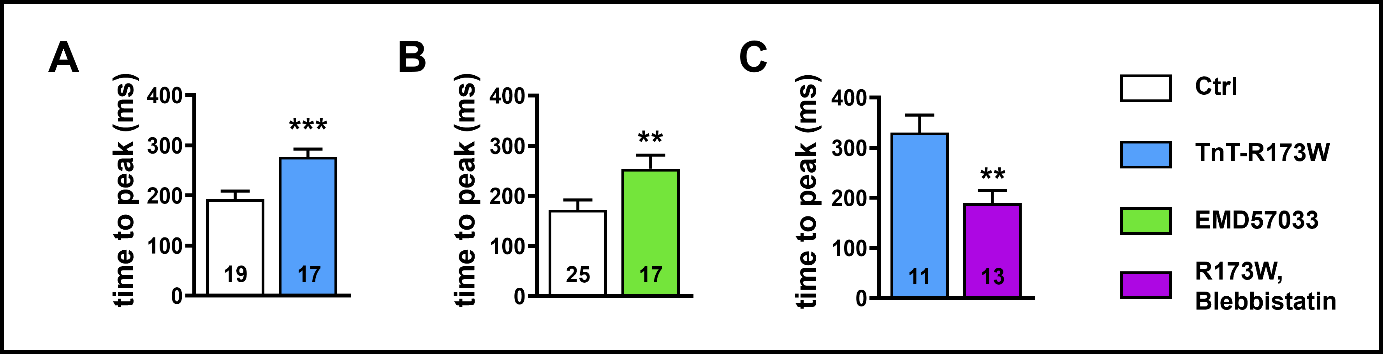
^

**Supplemental Figure 4:** **Increased time to peak in field stimulation triggered Ca^2+^-transients in induced pluripotent stem-cell derived cardiom­yocytes (iPSC-CM) with increased cytosolic calcium buffering.****A, B, C,** Ca^2+^ transient time to peak assigned to experiments shown in **Figure 2, 7, 8**. Time-to-peak values were determined by measuring the time between electrical stimulation and maximum Ca^2+^ level in an averaged trace generated from 10 consecutive traces. n = number of iPSC-CM from 2-5 batches. Data are mean±SEM. **P < 0.01 vs. Ctrl using Student’s t-test (**A, C**) and the Mann-Whitney U test (**B**).


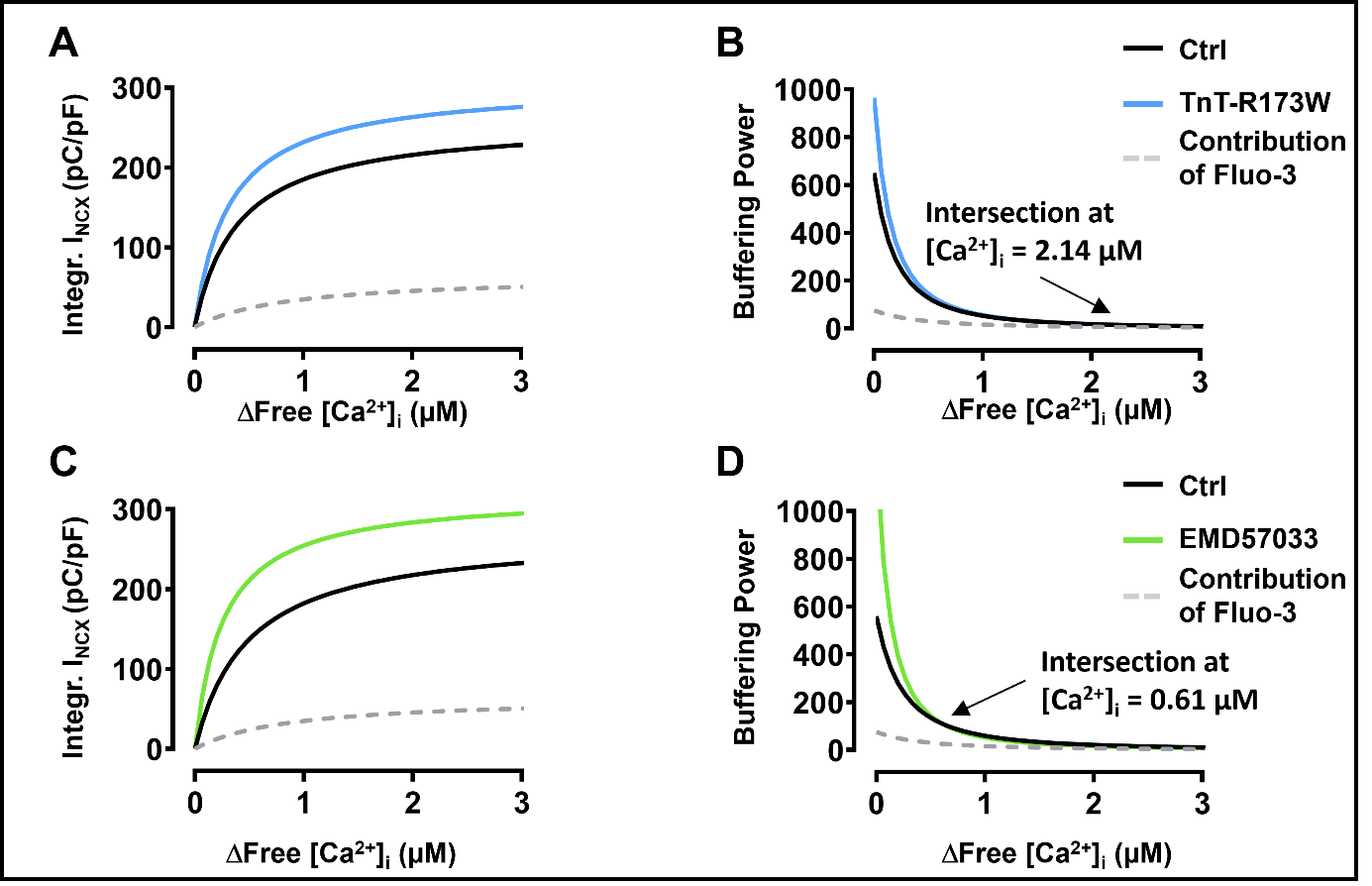


**Supplemental Figure 5: Buffer curves and buffer power in control (Ctrl), DCM-TnT-R173W and Ctrl pre-treated with EMD57033 induced pluripotent stem-cell derived cardiomyocytes (iPSC-CM).
A,** Cytosolic buffer curves shown as a plot of integrated I_NCX_ against a wide range of [Ca^2+^]_i_ created using the experimental estimated means of *K_d_* and *B_max_* values from Ctrl and TnT-R173W iPSC-CM. **B,** Buffering power, i.e. the change of [Ca^2+^]_total_ divided by the change of [Ca^2+^]_i_ (d[Ca^2+^]_total_/d[Ca^2+^]_i_) as a function of [Ca^2+^]_i_ estimated from the mean buffer curves in Ctrl and TnT-R173W iPSC-CM (**A**). The arrows indicate the calculated point of intersection of two buffer power curves. **C**, Cytosolic buffer curves in Ctrl iPSC-CM and Ctrl iPSC-CM pre-treated with EMD57033. **D**, Buffering power of Ctrl iPSC-CM and Ctrl iPSC-CM pre-treated with EMD57033. Contribution of Fluo-3 to all buffer- and buffer power curves are illustrated as grey dashed lines.


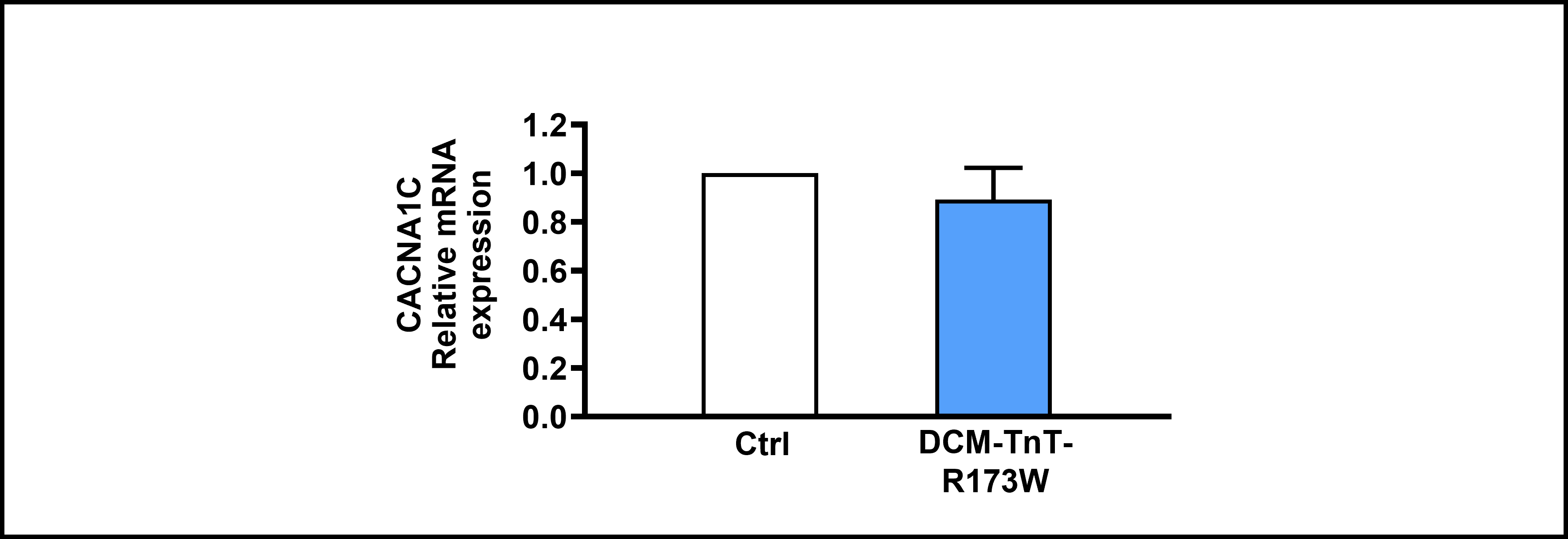


**Supplemental Figure 6: mRNA expression of Cav1.2 is not significantly altered in DCM-TnT-R173W iPSC-CM vs. control**Relative mRNA expression of Cav1.2 (CACNA1C) was assessed with quantitative real-time PCR (qRTPCR) in DCM patient-derived (DCM-TnT-R173W) iPSC-CM and Control (Ctrl) iPSC-CM. n = 3 (independent experiments). Data are mean±SEM. P = 0.674 using Student´s t-test.


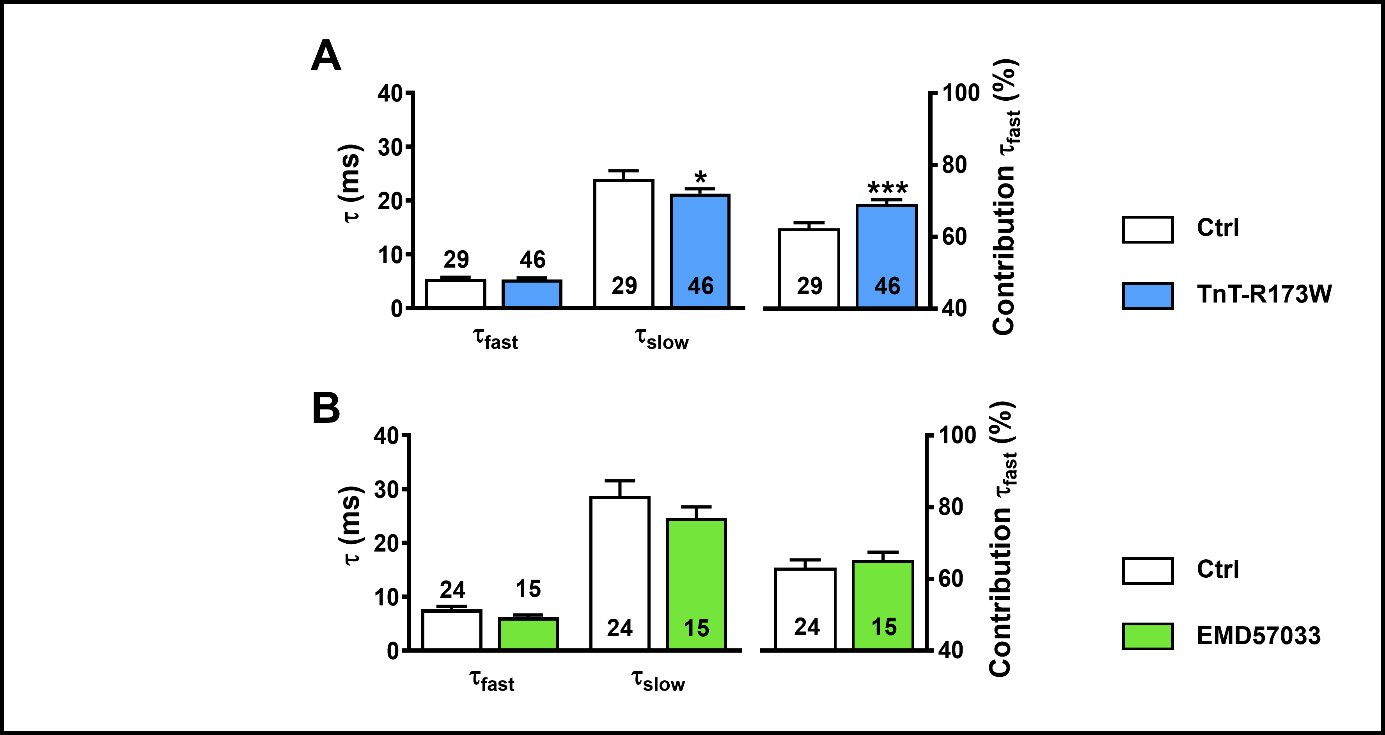


**Supplemental Figure 7:** **Further analysis of the biphasic I_Ca,L_ inactivation in control (Ctrl), DCM-TnT-R173W and Ctrl pre-treated with EMD57033 induced pluripotent stem-cell derived cardiomyocytes (iPSC-CM).**

**A, B,** Time constant of the fast part of the inactivation (τ_fast_, left), time constant of the slow part of inactivation (τ_slow_, middle), and the contribution of τ_fast_ to complete inactivation (%, right). n = number of iPSC-CM from 3-5 batches. Data are mean±SEM. *P < 0.05 and ***P < 0.001 vs. Ctrl using Mann-Whitney U test (**A, B left and middle**) and Student’s t-test (**B right**).

**
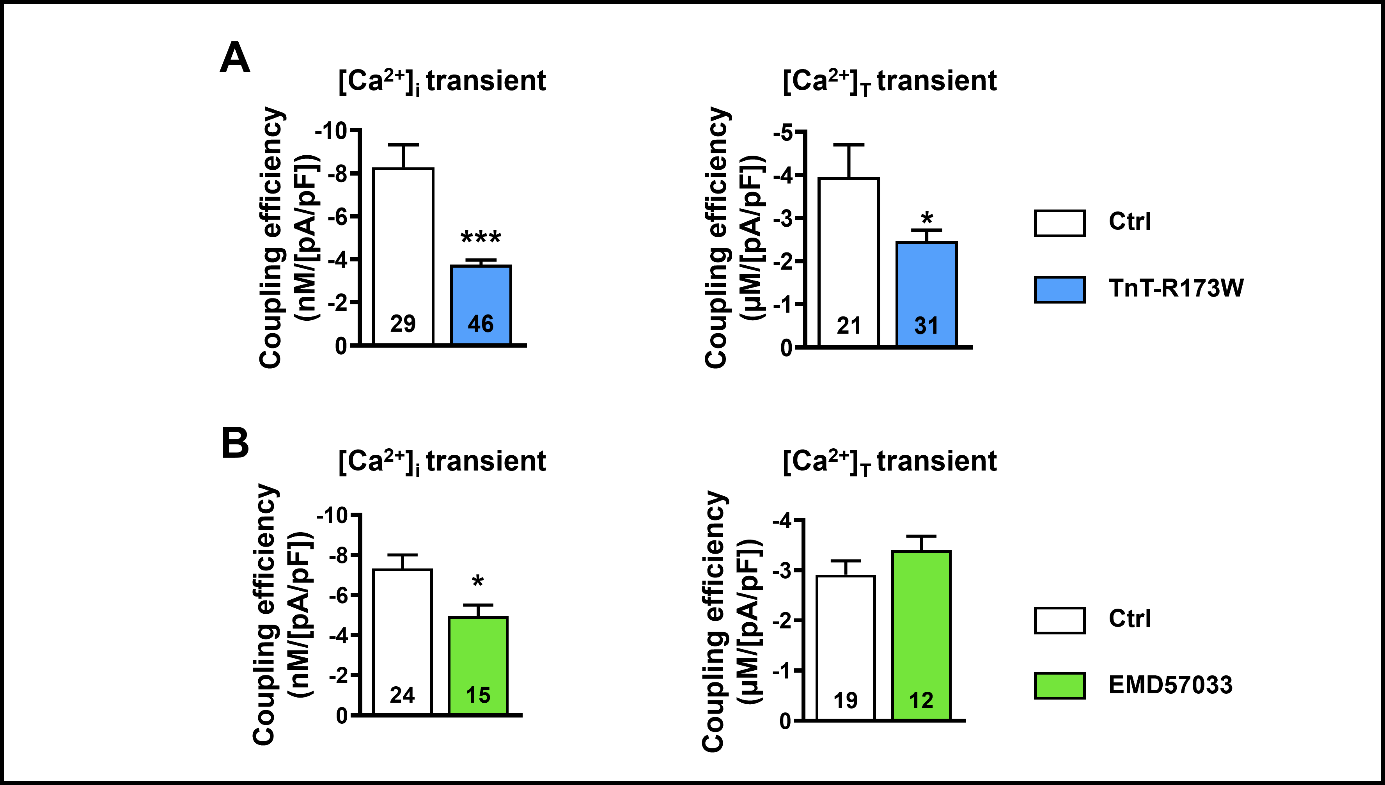
**

**Supplemental Figure 8:** **Coupling efficiency in control (Ctrl), DCM-TnT-R173W and Ctrl pre-treated with EMD57033 induced pluripotent stem-cell derived cardiomyocytes (iPSC-CM).**

**A, B,** “coupling efficiency” of Ca^2+^ influx and sarcoplasmic reticulum Ca^2+^ release estimated with experimental measured free Ca^2+^ ([Ca^2+^]­­_i_, left­) and calculated total Ca^2+^ ([Ca^2+^]­­_T_, right). n = number of iPSC-CM from 3-5 batches. Data are mean±SEM. *P < 0.05 and ***P < 0.001 vs. Ctrl using Mann-Whitney U test.


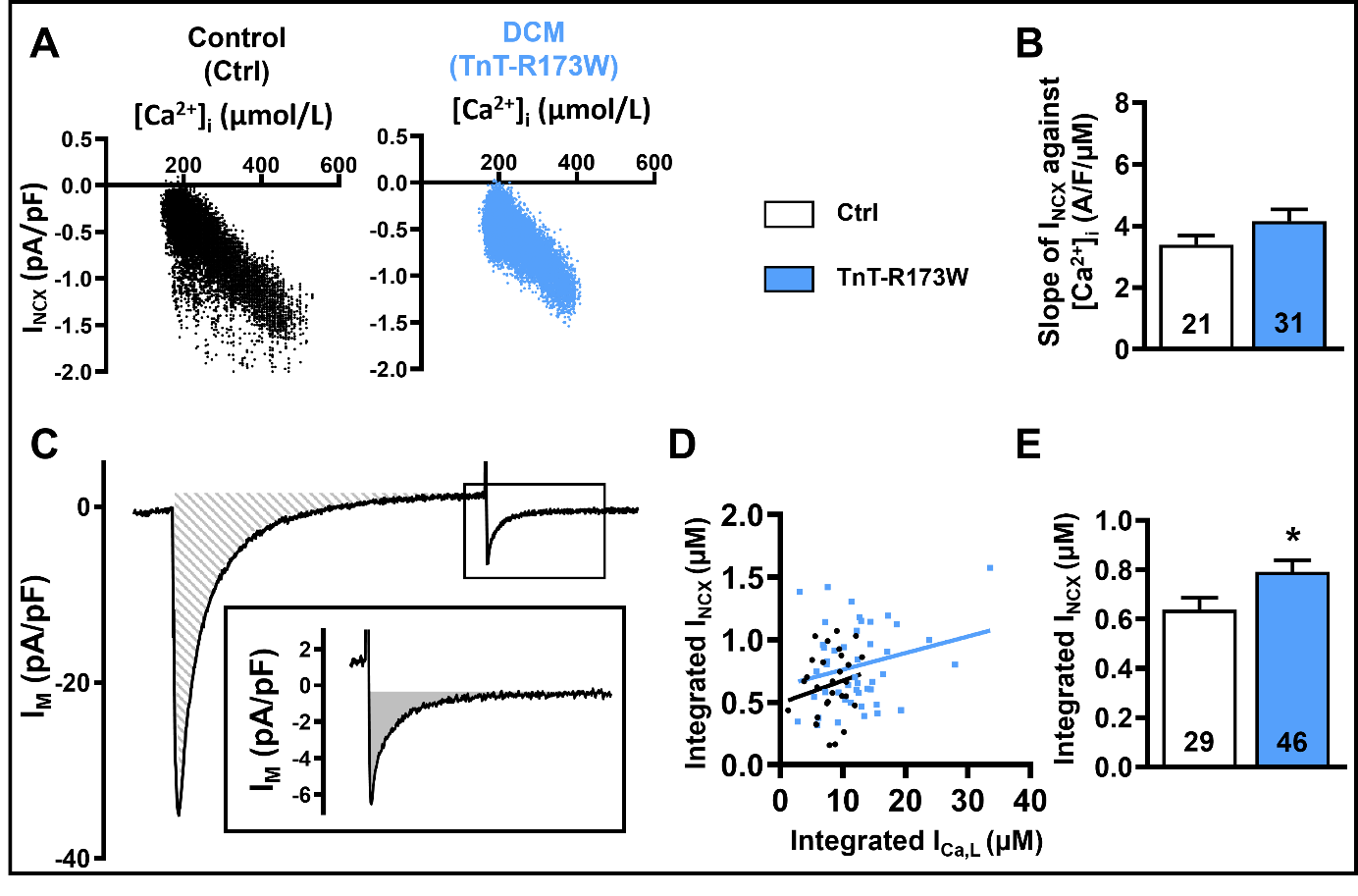


**Supplemental Figure 9: Comparable NCX function between control (Ctrl) and DCM-TnT-R173W induced pluripotent stem-cell derived cardiomyocytes (iPSC-CM).**

**A,** I_NCX_ in relation to [Ca^2+^]_i_ during decay of caffeine-induced Ca^2+^ transient from representative experiments using Ctrl (left) and TnT-R173W (right) iPSC-CM. **B,** The slope of a fitted linear function using I_NCX_ against [Ca^2+^]_i_ (**A**) is indicating the [Ca^2+^]_i_-dependence of NCX function. **C,** Representative membrane current according to voltag-e-clamp protocol showing I_Ca,L_ (left) and I_NCX_ (right, framed). The striped area visualises the integrated I_Ca,L_ representing the charge of Ca^2+^ entering the cell during stimulation (**Figure 3B**). The grey area visualises the integrated I_NCX_ representing the charge leaving the cell through the NCX after stimulation. **D,** Integrated I_NCX_ plotted against integrated I_Ca,L_ and correlated by a linear regression. Slope of Ctrl = 0.018, slope of DCM-TnT-R173W = 0.013. **E,** Integrated I_NCX_. n = number of iPSC-CM from 3-5 batches. Data are mean±SEM. *P < 0.05 vs. Ctrl using Mann-Whitney U test (**B**) and Student’s t-test (**E**).

**Supplemental References**

1. Chen G, Gulbranson DR, Hou Z, Bolin JM, Ruotti V, Probasco MD, Smuga-Otto K, Howden SE, Diol NR, Propson NE, Wagner R, Lee GO, Antosiewicz-Bourget J, Teng JMC, Thomson JA (2011) Chemically defined conditions for human iPSC derivation and culture. Nat Methods 8:424–429. doi: 10.1038/nmeth.1593

2. Dai Y, Amenov A, Ignatyeva N, Koschinski A, Xu H, Soong PL, Tiburcy M, Linke WA, Zaccolo M, Hasenfuss G, Zimmermann W-H, Ebert AD (2020) Troponin destabilization impairs sarcomere-cytoskeleton interactions in iPSC-derived cardiomyocytes from dilated cardiomyopathy patients. Sci Rep 10:209. doi: 10.1038/s41598-019-56597-3

3. Ebert AD, Kodo K, Liang P, Wu H, Huber BC, Riegler J, Churko J, Lee J, Almeida P de, Lan F, Diecke S, Burridge PW, Gold JD, Mochly-Rosen D, Wu JC (2014) Characterization of the molecular mechanisms underlying increased ischemic damage in the aldehyde dehydrogenase 2 genetic polymorphism using a human induced pluripotent stem cell model system. Science translational medicine 6:255–130. doi: 10.1126/scitranslmed.3009027

4. Fakuade FE, Steckmeister V, Seibertz F, Gronwald J, Kestel S, Menzel J, Pronto JRD, Taha K, Haghighi F, Kensah G, Pearman CM, Wiedmann F, Teske AJ, Schmidt C, Dibb KM, El-Essawi A, Danner BC, Baraki H, Schwappach B, Kutschka I, Mason FE, Voigt N (2021) Altered atrial cytosolic calcium handling contributes to the development of postoperative atrial fibrillation. Cardiovasc Res 117:1790–1801. doi: 10.1093/cvr/cvaa162

5. Lan F, Lee AS, Liang P, Sanchez-Freire V, Nguyen PK, Wang L, Han L, Yen M, Wang Y, Sun N, Abilez OJ, Hu S, Ebert AD, Navarrete EG, Simmons CS, Wheeler M, Pruitt B, Lewis R, Yamaguchi Y, Ashley EA, Bers DM, Robbins RC, Longaker MT, Wu JC (2013) Abnormal calcium handling properties underlie familial hypertrophic cardiomyopathy pathology in patient-specific induced pluripotent stem cells. Cell Stem Cell 12:101–113. doi: 10.1016/j.stem.2012.10.010

6. Pearman CM (2014) An Excel-based implementation of the spectral method of action potential alternans analysis. Physiol Rep 2:e12194. doi: 10.14814/phy2.12194

7. Sun N, Yazawa M, Liu J, Han L, Sanchez-Freire V, Abilez OJ, Navarrete EG, Hu S, Wang L, Lee A, Pavlovic A, Lin S, Chen R, Hajjar RJ, Snyder MP, Dolmetsch RE, Butte MJ, Ashley EA, Longaker MT, Robbins RC, Wu JC (2012) Patient-specific induced pluripotent stem cells as a model for familial dilated cardiomyopathy. Sci Transl Med 4:130ra47. doi: 10.1126/scitranslmed.3003552

8. Wu H, Lee J, Vincent LG, Wang Q, Gu M, Lan F, Churko JM, Sallam KI, Matsa E, Sharma A, Gold JD, Engler AJ, Xiang YK, Bers DM, Wu JC (2015) Epigenetic Regulation of Phosphodiesterases 2A and 3A Underlies Compromised β-Adrenergic Signaling in an iPSC Model of Dilated Cardiomyopathy. Cell Stem Cell 17:89–100. doi: 10.1016/j.stem.2015.04.020
